# Changes in membrane sphingolipid composition modulate dynamics and adhesion of integrin nanoclusters

**DOI:** 10.1038/srep20693

**Published:** 2016-02-12

**Authors:** Christina Eich, Carlo Manzo, Sandra de Keijzer, Gert-Jan Bakker, Inge Reinieren-Beeren, Maria F. García-Parajo, Alessandra Cambi

**Affiliations:** 1 Department of Tumor Immunology, Radboud Institute for Molecular Life Sciences, Radboud University Medical Center, Postbox 9101, 6500 HB Nijmegen, The Netherlands; 2 ICFO-Institut de Ciencies Fotoniques, Mediterranean Technology Park, 08860 Castelldefels (Barcelona), Spain; 3 Department of Cell Biology, Radboud Institute for Molecular Life Sciences, Radboud University Medical Center, Postbox 9101, 6500 HB Nijmegen, The Netherlands; 4 ICREA-Institució Catalana de Recerca i Estudis Avançats, 08010 Barcelona, Spain

## Abstract

Sphingolipids are essential constituents of the plasma membrane (PM) and play an important role in signal transduction by modulating clustering and dynamics of membrane receptors. Changes in lipid composition are therefore likely to influence receptor organisation and function, but how this precisely occurs is difficult to address given the intricacy of the PM lipid-network. Here, we combined biochemical assays and single molecule dynamic approaches to demonstrate that the local lipid environment regulates adhesion of integrin receptors by impacting on their lateral mobility. Induction of sphingomyelinase (SMase) activity reduced sphingomyelin (SM) levels by conversion to ceramide (Cer), resulting in impaired integrin adhesion and reduced integrin mobility. Dual-colour imaging of cortical actin in combination with single molecule tracking of integrins showed that this reduced mobility results from increased coupling to the actin cytoskeleton brought about by Cer formation. As such, our data emphasizes a critical role for the PM local lipid composition in regulating the lateral mobility of integrins and their ability to dynamically increase receptor density for efficient ligand binding in the process of cell adhesion.

In the modern view of the plasma membrane (PM), protein-protein, protein-lipid and lipid-lipid interactions occur in a dynamic fashion and lead to local segregation into PM compartments that are important to regulate signal transduction^1^,^2^. The best described PM compartments are the so called “lipid rafts” that are rich in cholesterol, glycosphingolipids, sphingomyelin (SM) and embed raftophilic proteins such as glycosylphosphatidyl-inositol anchored proteins (GPI-APs)^2^. Advances in microscopy techniques now allow direct visualisation of PM lipid nanodomains, such as those consisting of the glycosphingolipids GM1^3^, GM3^4^, SM^5^ and PIP2^6^. More recently, nanoscopy approaches have captured fast molecular movements of individual PM lipids in living cells revealing heterogeneous mobility behaviours including transient trapping of sphingolipids in cholesterol-mediated molecular complexes^7^,^8^,^9^.

The specific lipid nanoenvironment in which PM proteins are embedded seems crucial in regulating receptor function. So is the activation state of an ion channel directly modified by its surrounding lipids^10^, and the allosteric transition of the epidermal growth factor receptor from an inactive to an active signalling dimer regulated by interaction with GM3^11^. Glycosphingolipids have also been implicated in providing membrane platforms facilitating to the formation of toxic amyloid-beta structures eventually leading to membrane fragmentation^12^,^13^. Similarly, cholesterol locally sequesters proteins involved in signal transduction^14^ or induces conformational changes of glycolipid headgroups, thereby modulating properties of bioactive glycolipids^15^. Also of interest is the role of cholesterol in modulating the selectivity of antimicrobial peptides for bacterial membranes, role that appears modulated by the localization of cholesterol into lipid rafts^16^,^17^,^18^.

Strikingly, the cellular levels of (glyco)-sphingolipids and cholesterol as well as the expression of lipid metabolizing enzymes are altered in a variety of diseases including cancer^19^,^20^, in response to external stimuli such as pathogens^21^ or induced by drug treatment^22^. For example, modification of PM lipids by sphingomyelinase (SMase) is highly relevant *in vivo*, as triggering of human dendritic cells by Measle virus induces activation of SMase that locally alters the PM and further promotes virus uptake in these cells^23^. SMase induces breakdown of SM into Ceramide (Cer), which partially displaces cholesterol from rafts^24^,^25^ and leads to the formation of large Cer-enriched membrane domains in model membranes^26^,^27^. Importantly, formation and dynamics of Cer-enriched membrane platforms has been also documented in living cells^28^, where they can influence function and avidity state of several receptors^21^. For example, CD95 triggers apoptosis upon ceramide-induced cluster formation^29^ while ceramide platforms facilitate interactions between CD38 and the muscarinic type 1 (M(1)) receptor required for induction of intracellular cyclic ADP-ribose^30^ and locally promote the translocation of the transferrin receptor to clathrin-coated pits for subsequent endocytosis^31^. It is therefore plausible that the function and dynamics of many other transmembrane (TM) receptors are likely to be influenced by *in vivo* dynamic changes of lipid content in response to environmental cues.

Integrins are TM receptors that mediate cell-cell and cell-matrix interactions and play a key role during cell adhesion and migration. An α and a β subunit form a functional heterodimer, and their regulation occurs via conformational changes that alter affinity for their ligands^32^ or via dynamic redistribution within the membrane that locally increases the receptor density (i.e. valency) leading to increased avidity^33^. The hydrophobic TM regions of integrins have been recently shown to undergo important conformational changes that seem crucial in regulating integrin signalling^34^. These findings might challenge the classical protein-focused view on integrin regulation solely mediated by affinity and avidity mechanisms. Since the TM regions are in direct contact with the lipid nanoenvironment of the PM bilayer, it is very likely that changes in the local lipid composition can impact on integrin regulation. Indeed, an earlier study by Feldhaus and colleagues^35^ indicated that Cer generation by SMase impaired β2 integrin-mediated adhesion, although the underlying molecular mechanisms for this inhibitory effect remain so far unknown.

*Lymphocyte function-associated antigen 1* (LFA-1, αLβ2) is a leukocyte specific integrin that mediates firm arrest on the endothelium and within the lymph nodes, cell tethering and the formation of the immunological synapse^36^. By exploiting high resolution imaging techniques, we showed that LFA-1 on quiescent monocytes is organized in well-defined nanoclusters^37^ that reside in nanoscale proximity to domains enriched in GPI-APs or GM1, without physical intermixing^3^,^38^. Importantly, we demonstrated an essential role of cholesterol in mediating LFA-1-GPI-AP interactions at the nanoscale and in the formation of larger raft-based adhesion sites upon ligand binding^3^,^38^. Furthermore, we applied single particle tracking (SPT) and showed that lateral diffusion and conformation states of LFA-1 nanoclusters are highly interlinked: LFA-1 was mainly mobile, with slow and fast diffusion profiles, while a small sub-population of high-affinity LFA-1 was immobilized by anchorage to the cytoskeleton^39^. While several studies have reported a dependency of TM receptor mobility on the PM cholesterol, Cer and SM content^40^,^41^,^42^, it is still unknown whether these lipids contribute to the regulation of LFA-1 mobility and whether LFA-1 function is sensitive to changes in PM lipid composition.

In this study we investigated how the lipid nano-environment regulates LFA-1 function and mobility by reducing the SM content at the PM by myriocin or by conversion of SM into Cer by SMase. Our work demonstrates that SM conversion into Cer influences LFA-1 function and lateral mobility, suggesting the involvement of the cortical actin cytoskeleton. By combining SPT and biochemical assays, our results reveal that PM lipid alteration by induction of SMase activity can negatively affect integrin function by compromising lateral mobility, ultimately interfering with leukocyte adhesion.

## Results

### LFA-1 binding capacity and proximity to GM1 enriched domains are sensitive to the SM content in the plasma membrane

**Figure 1:**
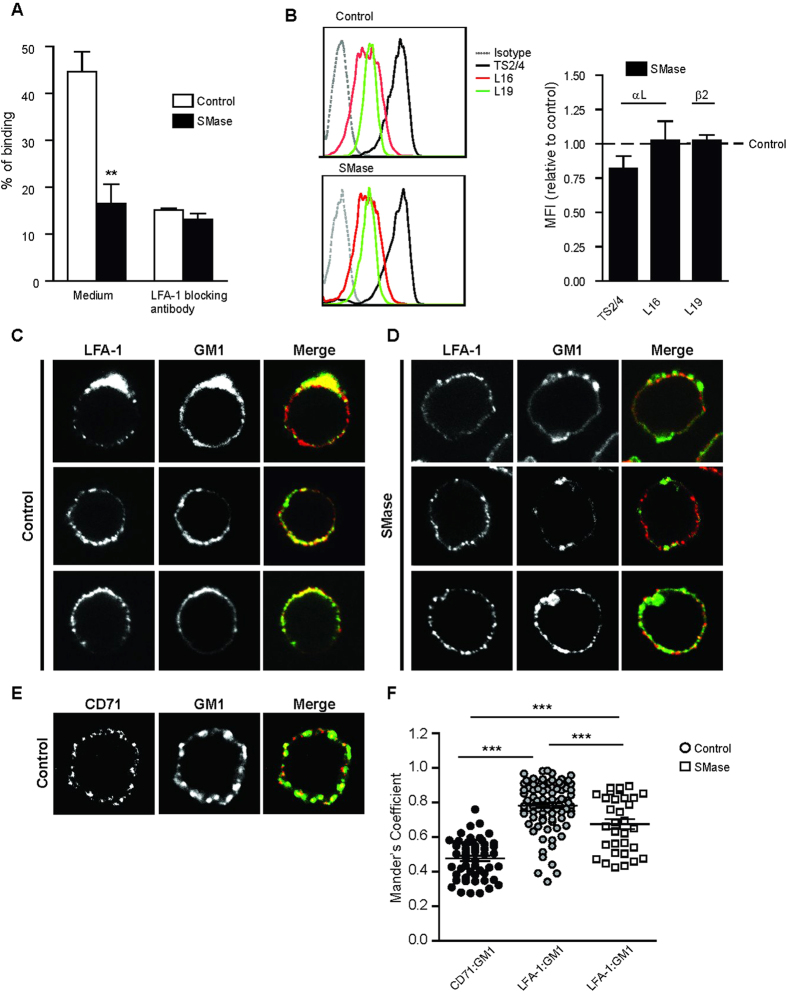
LFA-1 binding capacity on monocytes and proximal distribution to GM1 is reduced by SMase. **(A**) Adhesion of human monocytes (THP-1) was determined using ICAM-1-Fc coated fluorescent beads at 37 °C in unperturbed cells and upon treatment with SMase (0.05 U/ml). The % of adhesion represents the amount of cells that have bound beads as determined by flow cytometry. NKI-L15 mAb was used to block LFA-1. The data shows one representative experiment of 4 ± SD. **(B**) Relative expression of two αL–(TS2/4 and L16) and one β2-(L19) specific epitope after treatment with SMase, assessed by flow cytometry. Left, representative FACS profiles in control cells and upon treatment with SMase. Right, changes in the mean fluorescent intensity are displayed as relative to control sample (expression levels in unperturbed cells were set as = 1, indicated by the dotted line). The data represent the mean ± SEM of 5–7 independent experiments. **(C**) Confocal microscopy analysis of co-capping of LFA-1 (L15 labeled) and GM1 (CTx-AF647) in untreated cells. (**D**) Confocal microscopy analysis of co-capping of LFA-1 (L15 labeled) and GM1 (CTx-AF647) in SMase treated cells. Images depict three representative cells for each condition. (**E**) Confocal microscopy analysis of co-capping of CD71 and GM1 (CTx-AF647) in untreated cells. Receptor co-capping and staining were performed as described in *Material and Methods*. (**F**) Colocalization between LFA-1 or CD71 and GM1 as determined by the Manders coefficients (M1). Results are representative of multiple cells in three independent experiments. All P-values were compared to the respective unperturbed cells by 1way ANOVA with Student Neuman–Keuls post-test, ***<0.001.

It has been previously shown that a decrease in SM levels on the PM negatively affected β_2_ integrin activity in neutrophils^35^. Moreover, using super-resolution microscopy we demonstrated that LFA-1 binding to its ligand ICAM-1 on monocytes depended on cholesterol and its spatial proximity to GM1 and GPI-AP nanodomains^3^,^38^. These findings prompted us to investigate how local changes in other lipid raft components, such as SM, would affect LFA-1 lateral organization and binding to ICAM-1. To understand how sensitive LFA-1 function is to local changes in SM, we determined the binding of monocytes to ICAM-1-Fc coated fluorescent beads by flow cytometry after converting endogenous SM into Cer by recombinant SMase (Fig. 1A). In agreement with previous results^37^, around 40% of unperturbed monocytes spontaneously bound ICAM-1, and this interaction was specifically LFA-1 mediated, as shown by the effective blocking in the presence of an anti- αL mAb. In contrast, conversion of SM into Cer by SMase addition reduced the binding to ICAM-1 to less than 20%, as effectively as the blocking mAb. Of note, SMase was applied at optimal concentrations that preserved cell viability, but significantly reduced the SM content at the membrane by efficiently inducing SM conversion into Cer, without affecting cholesterol or GM1 levels (Supplementary Fig. S1A–C). To note, SMase washout did not restore LFA-1 adhesion to ICAM-1 coated beads suggesting that the blocking effect of Cer formation remains for some time (Supplementary Fig. S1D), as also observed for other membrane receptors^43^.

To enquire whether the reduction in binding to ICAM-1 resulted from a decrease in LFA-1 cell surface expression and/or a change in LFA-1 affinity state in response to SMase treatment, we determined the binding of the αL specific neutral mAb TS2/4, the extension reporter antibody NKI-L16^44^, as well as the mAb L19 recognizing the integrin β_2_ chain, before and after SMase treatment (Fig. 1B). Interestingly, we did not observe significant changes in expression levels of total LFA-1 or β_2_ integrins with respect to unperturbed cells, neither changes in the expression levels of extended LFA-1. Altogether, these results indicate that SMase treatment impairs LFA-1-mediated adhesion in monocytes, without affecting LFA-1 cell surface expression levels or the degree of LFA-1 activation, as reported by the NKI-L16 Ab.

To investigate whether altered SM levels had an overall effect on the co-distribution of LFA-1 and the raft-associated GM1, we performed antibody-induced capping and confocal microscopy on untreated and SMase treated cells. On SMase treated cells, the co-distribution of LFA-1 and GM1 patches was visually reduced compared to untreated cells (Fig. 1C,D). However, the integrity of LFA-1 nanoclusters remained unaffected by the treatment, as shown by Electron Microscopy images of whole-mount cells labelled for LFA-1 with 10 nm gold particles (Supplementary Fig. S2). The average Manders co-localization coefficient of the non-raft marker CD71 with GM1 (Fig. 1E) was 0.46, while raft-associated LFA-1 with GM1 in unperturbed cells showed a significantly higher co-localization coefficient of about 0.8 (Fig. 1F). In contrast, upon SMase treatment co-localization between LFA-1 and GM1 was significantly reduced (SMase = 0.68), suggesting an increasing exclusion of LFA-1 from GM1 enriched domains upon SM reduction and conversion to Cer. These results thus suggest that LFA-1 ligand binding and its association with raft components depend on the SM content in the PM of monocytes.

### Conversion of SM into Cer alters LFA-1 lateral mobility

**Figure 2:**
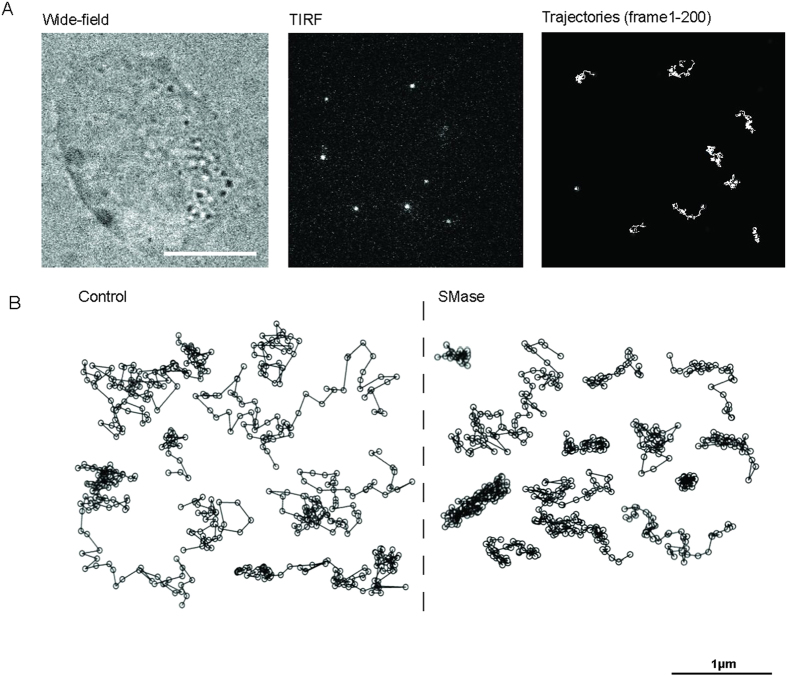
Trajectories of LFA-1 nanoclusters are visually affected by SMase. (**A**) Widefield image of a living THP-1 cell (left) and selected frame from a movie recorded at 100ms/frame in TIRF mode (middle). Bright spots correspond to individual LFA-1 nanoclusters labeled with TS2/4-Atto647N. Examples of different trajectories as they are tracked on the cell membrane of THP-1 cells over 200 frames (right). Scale bar: 10 μm. (**B**) Representative trajectories of LFA-1 nanoclusters in unperturbed (left) and SMase (right) treated cells, illustrating different lateral mobilities.

It has been extensively shown that the mobility of transmembrane receptors depends on the local PM environment^41^,^42^,^45^. Since SMase treatment decreased the overall content of SM with a concomitant increase of Cer production at the PM, we sought to investigate how changes in the lipid nano-environment as those induced by SMase affect LFA-1 lateral mobility. Individual LFA-1 nanoclusters labeled with the neutral mAb TS2/4-Atto647N (sub-labeling conditions) were imaged in the ventral PM using a TIRF geometry (Fig. 2A) and individual trajectories were subsequently generated. Consistent with our previous results, LFA-1 trajectories in unperturbed monocytes showed diverse mobility, ranging from highly mobile to restricted and immobile (Fig. 2B)^39^. Treatment with SMase led to visually more restricted trajectories (Fig. 2B).

**Figure 3:**
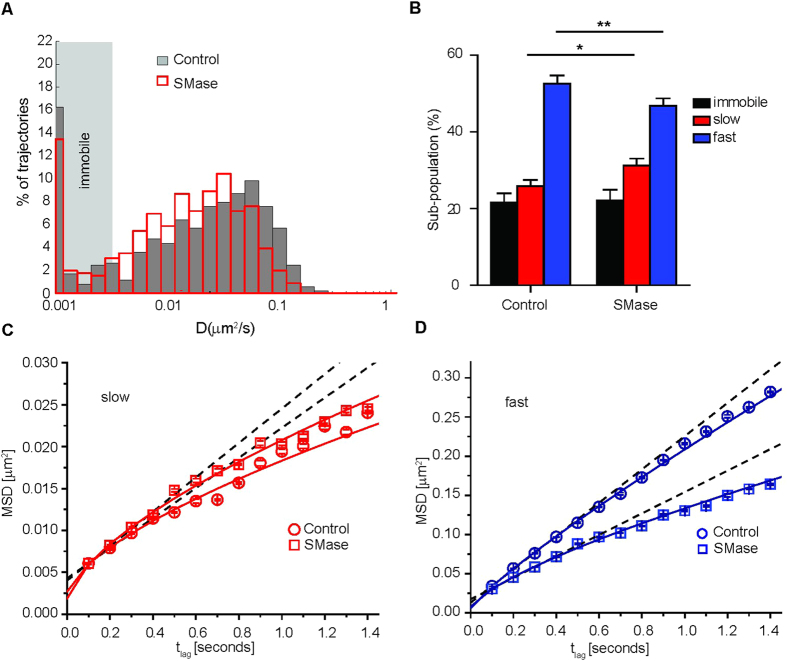
SMase influences the lateral mobility of LFA-1 nanoclusters. **(A**) Normalized semilog distribution of D_1–4_ values for LFA-1 nanoclusters in unperturbed control cells (grey) or after SMase (red) treatment. Each histogram contains at least 400 trajectories taken from >30 cells in 5 experiments. The vertical dotted lines represent the average *D*_1–4_ values in control cells (black) and SMase treated cells (red). (**B**) Percentage of LFA-1 nanocluster mobility classified as immobile, slow and fast mobile in unperturbed and SMase treated monocytes. The error bars represent the mean sub-population size ± SEM of 5 independent experiments. 2way ANOVA and Bonferroni post-test were applied to the full distribution of sub-population values calculated from at least 30 cells in 3–5 experiments. P-values were compared to the respective unperturbed cells, *<0.05, **<0.01. **(C,D**) Square displacement plots of the slow (**C**) and fast (**D**) moving population of LFA-1 by fitting the CPD at different time lags (461–658 trajectories). Slow and fast diffusing components were fitted to a model of i) free (dashed lines) and ii) anomalous diffusion (continuous lines).

To quantify differences in the nanoscale diffusion behavior of LFA-1 nanoclusters, we analyzed individual trajectories by calculating the mean squared displacement (MSD) as a function of the time lag and generated histograms of the short range diffusion coefficients (*D*_1–4_ values) (see methods) (Fig. 3A). In unperturbed monocytes the *D*_1–4_ values varied from 0.0046 μm^2^/s (minimum detectable *D*_1–4_ value for mobile trajectories, see materials and methods section and supplementary Fig. S3) to 0.4 μm^2^/s, with an average *D*_1–4_ = 0.04 ± 0.0001 μm^2^/s and with ~22% of immobile nanoclusters (Fig. 3A,B). SMase treatment led to a shift of the *D*_1–4_ distribution to lower values with a two-fold decrease in the average *D*_1–4_ (0.024 ± 0.0002 μm^2^/s), while the percentage of immobile nanoclusters was not significantly affected (Fig. 3A,B). To exclude the possibility that SMase treatment induced unwanted global changes in PM fluidity that could explain the reduction in LFA-1 mobility, we used fluorescence recovery after photobleaching to measure the mobility of the PKH dye, a lipid probe that inserts into the lipid bilayer. The results showed no difference in recovery of the PKH dye upon SMase treatment with respect to unperturbed cells (Supplementary Fig. S4), indicating no global fluidity changes on the PM that could explain the changes on LFA-1 mobility upon SMase treatment.

Since *D*_1–4_ only reports on the short-time diffusion behaviour, we performed cumulative probability distribution (CPD) analysis on the ensemble of mobile trajectories to obtain information about the long-time (>1 s) diffusion behavior of the mobile LFA-1 population (see *Materials and Methods*). Using this approach, we could discriminate a slow and a fast diffusing sub-population, and calculate their relative sizes (Fig. 3B). Notably, SMase treatment significantly increased the slow mobile sub-population from 26 ± 2% to 31 ± 2% and decreased the fast mobile sub-population from 53 ± 2% to 46 ± 2% (Fig. 3B).

The mobility was compared by plotting the CPD square displacement values against the respective time lags for the slow (Fig. 3C) and fast mobile (Fig. 3D) sub-populations in unperturbed and SMase-treated monocytes. The slow mobile sub-population was not significantly affected by the treatment (Fig. 3C and Table 1). In contrast, the square displacement plots of the fast mobile sub-population showed a remarkable reduction in the diffusion (from *D*_f_ = 0.053 μm^2^/s in unperturbed cells to *D*_f_ = 0.035 μm^2^/s in SMase treated cells) and an increase in the anomalous diffusion (from α = 0.86 on unperturbed to α = 0.74 on SMase treated cells) in response to SMase treatment (Fig. 3D and Table 1).

**Figure 4:**
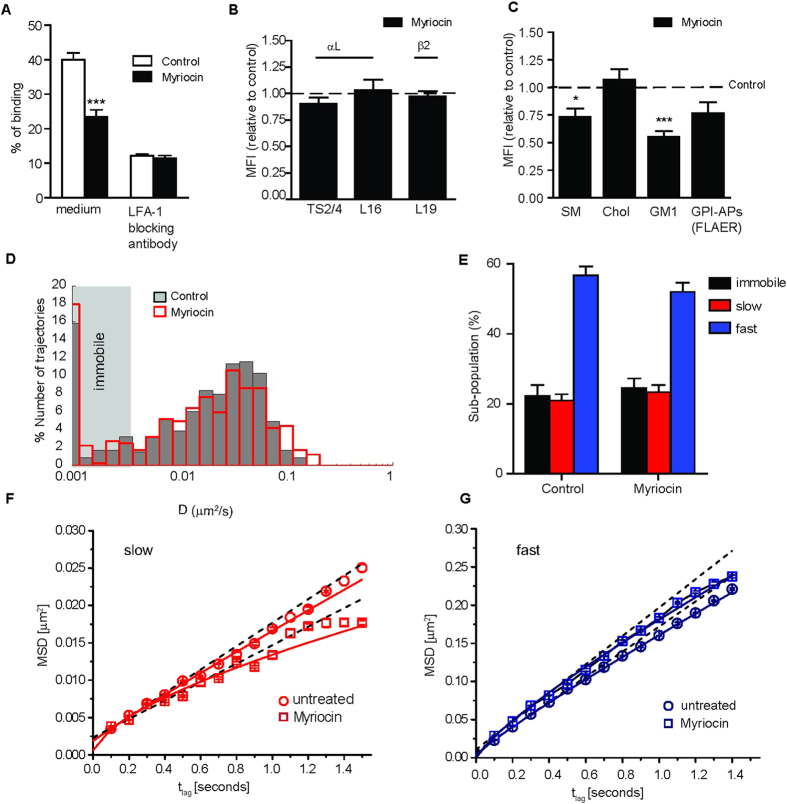
Metabolic depletion of SM reduces LFA-1 adhesion without affecting its lateral mobility. (**A**) Monocyte adhesion to ICAM-1-Fc-coated fluorescent beads at 37 °C in unperturbed and myriocin-treated cells. The % of adhesion represents the amount of cells that have bound beads as determined by flow cytometry. NKI-L15 mAb was used to block LFA-1. The data show one representative experiment of 4 ± SD. Differences were assessed by 1way ANOVA test, ***<0.001. **(B**) Relative expression of αL– and β2- specific epitopes were assessed by flow cytometry. Mean fluorescent intensity changes are displayed as relative to control sample (expression levels in unperturbed cells set as = 1, dotted line). The data represent the mean ± SEM of 5–7 independent experiments. **(C**) Total SM content and PM cholesterol levels were determined by lysenin and filipin III labelling, respectively, while PM GM levels were detected by Ctx-AF647 and PM GPI-APs were detected by FLAER. The relative expression was assessed by flow cytometry and mean fluorescent intensity changes are relative to the respective unperturbed sample (expression levels in untreated cells set as = 1, dotted line). The data represent the mean ± SEM of 3–5 independent experiments. Differences were assessed by 1way ANOVA test, *<0.05, ***<0.001. **(D**) Normalized semilog distribution of D_1–4_ values for LFA-1 nanoclusters in unperturbed control (grey) or myriocin-treated cells (red). Vertical dotted lines represent the average *D*_1–4_ values in control (black) and myriocin-treated cells (red). Each histogram contains at least 406 trajectories taken from 30 cells in 3–5 experiments. **(E**) Percentage of LFA-1 nanocluster mobility classified as immobile, slow and fast mobile in unperturbed and myriocin treated monocytes. The error bars represent the mean ± SEM of 3–5 independent experiments. 2way ANOVA and Bonferroni post-test were applied to the full distribution of sub-population values calculated from at least 30 cells in 3–5 experiments. **(F,G**) Square displacement plots of slow and fast moving LFA-1 populations by fitting the CPD at different time lags (461–658 trajectories). Slow and fast diffusing components were fitted to a model of i) free (dashed lines) and ii) anomalous (continuous lines) diffusion.

To understand whether the observed effects of SMase on LFA-1 function and diffusion in monocytes are due to the formation of Cer or to a global reduction of the PM SM levels, we repeated the experiments after lowering the SM content by inhibition of the sphingolipid synthesis with myriocin^46^. Similar to SMase, myriocin reduced the binding to ICAM-1 with respect to unperturbed cells (Fig. 4A), without affecting the expression levels of total LFA-1 or β2 integrins in general (Fig. 4B). Myriocin treatment did not affect cell viability (Supplementary Fig. S5), but significantly reduced the SM and GM1 content at the PM, with a similar trend for GPI-APs, without affecting cholesterol levels, in agreement with earlier findings^47^ (Fig. 4C). Moreover, myriocin reduced the co-localization of LFA-1 with GM1 from 0.78 to 0.63 in co-capping experiments (Supplementary Fig. S5), without affecting the integrity of LFA-1 nanoclusters (Supplementary Fig. S5).

Next we examined the effect of myriocin on the mobility of individual LFA-1 nanodclusters (Fig. 4D–G). Interestingly and in marked contrast to SMase, myriocin treatment did not significantly affect the *D*_*1–4*_ values of LFA-1 nanoclusters compared to untreated cells (Fig. 4D). Of note, myriocin is applied in the absence of serum (see *Materials and Methods*) and therefore the control condition represents serum-starved cells (see Table 2). Similarly, CPD analysis to characterize the long-term diffusion behaviour of LFA-1 nanoclusters showed no significant differences in size (Fig. 4E) or the mobility of the slow and fast moving sub-populations of LFA-1 compared to untreated cells (Fig. 4F,G and Table 2).

Since myriocin and SMase both reduce the SM content, but only SMase leads to formation of Cer, we conclude that Cer formation, rather than reduced SM levels, is responsible for slowing down of the fast sub-population of mobile LFA-1 nanoclusters, which also exhibited increased anomalous diffusion as indicated by the lower α value (Table 1). However, since these two drugs remove SM from the plasma membrane to a different extent, we cannot exclude that SMase is simply much more powerful in depleting SM than myriocin thus explaining its higher impact on LFA-1 dynamics and function.

Cer formation also reduced the sub-population of fast mobile LFA-1 nanoclusters and increased the slow mobile sub-population (Table 1). These data collectively indicate that the metabolic reduction of SM and other glycosphingolipids by myriocin as well as the patho-physiological activation of SMase with subsequent conversion of SM into Cer impact on LFA-1 function. The underlying molecular mechanisms are probably different, as Cer formation (but not myriocin) significantly hampers LFA-1 mobility.

### Cer formation increases the association of LFA-1 with cortical actin

**Figure 5:**
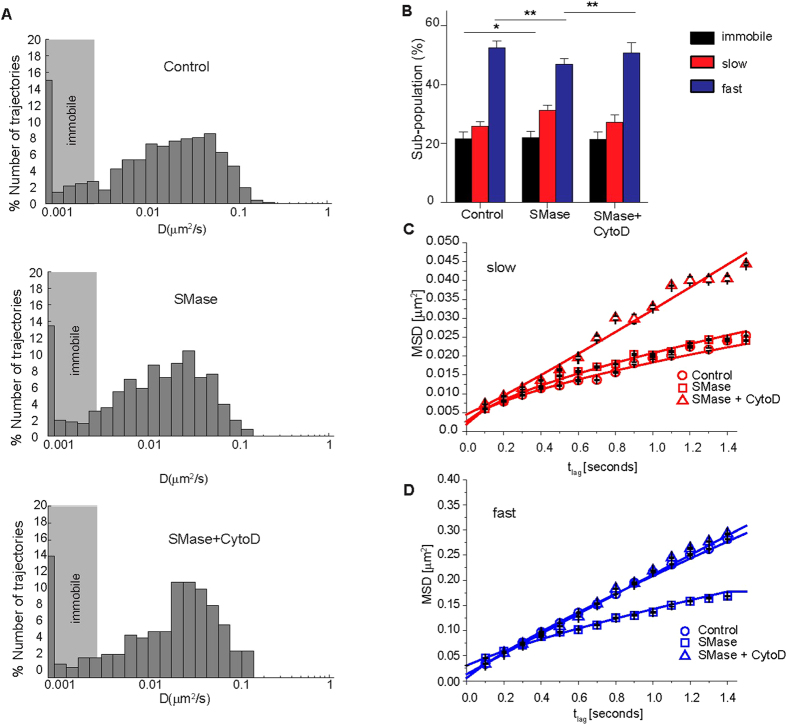
Cer induced reduction and restriction of LFA-1 diffusion involves cortical actin. (**A**) Normalized semilog distribution of (D_1–4_) values for LFA-1 nanoclusters in control cells (top), upon SMase treatment without (middle) or with (bottom) addition of 5ug/ml CytoD during measurements. Each histogram contains at least 262 trajectories taken from 30 cells in 3 experiments. (**B**) Percentage of LFA-1 mobility classified as immobile, slow and fast mobile in control cells, SMase-treated cells without or with addition of 5ug/ml CytoD during measurement. The error bars represent the mean ± SEM of 5 independent experiments. 2way ANOVA and Bonferroni post-test were applied to the full distribution of sub-population values calculated from at least 30 cells in 3–5 experiments, *<0.05, **<0.01. (**C,D**) CPD derived Square displacement plots of slow and fast moving LFA-1 nanoclusters in unperturbed cells (circle), SMase-treated cells without (square) or with addition of 5ug/ml CytoD (triangle) during measurement. Slow and fast diffusing components were fitted to a model of free and restricted/anomalous diffusion (see Table 1). The continuous line through the data points represents the fit of the anomalous diffusion model.

The extended form of LFA-1 has been shown to interact with cortical actin^39^,^48^ and actin has also been implicated in cholesterol-based PM nanoheterogeneity^49^,^50^, suggesting that the propensity of LFA-1 to interact with the cortical cytoskeleton and its proximal distribution to GM1-enriched domains and GPI-APs^3^,^38^ are interrelated. Moreover, it has been reported that Cer formation interferes with the dynamic organisation of the actin cytoskeleton^51^. Since we observed a reduction in LFA-1 mobility upon Cer formation, and integrins are known to interact with the cytoskeleton, we sought to measure the mobility of individual LFA-1 nanoclusters after SMase treatment and subsequent addition of the actin polymerisation inhibitor Cytochalasin D (CytoD). First, we plotted the D_1–4_ values versus time-lag of unperturbed, SMase treated cells, as well as of cells treated with SMase and CytoD (Fig. 5A). Remarkably, addition of CytoD to SMase treated cells increased the average D_1–4_ from 0.024 ± 0.0003 μm^2^/s to 0.039 ± 0.0004 μm^2^/s, similar to the average diffusion values observed in unperturbed cells (0.040 ± 0.0001 μm^2^/s, Fig. 5A).

CPD analysis revealed no significant change in the immobile sub-population of LFA-1 upon concomitant addition of CytoD and SMase compared to SMase treated cells, however the slow sub-population slightly decreased from 31 ± 1.8% to 27 ± 2.5% and shifted towards the fast moving sub-population, which significantly increased from 46 ± 1.9% to 51 ± 3.4% (Fig. 5B), suggesting that CytoD treatment reverses the changes in sub-population size observed upon SMase treatment closer to those observed in unperturbed cells.

Next, we plotted the CPD derived square displacement against the respective time lags for the slow and fast moving sub-populations of LFA-1 in unperturbed and SMase treated cells, and upon concomitant addition of CytoD and SMase (Fig. 5C,D). Addition of CytoD to SMase-treated cells strikingly released slow-diffusing LFA-1 nanoclusters from constrains promoting their free diffusion on the cell surface (with α_s_ going from 0.66 to 1.1) (Fig. 5C and Table 1). Moreover, actin disruption concomitant to Cer production almost completely abrogated the effects of SMase on the mobility of the fast LFA-1 population, with D_f_ and α_f_ values that became closer to those of fully unperturbed cells (Fig. 5D and Table 1). These results are interesting as they indicate that the slow population of LFA-1 nanoclusters is bound to the actin cytoskeleton and not affected by SMase treatment. Importantly, CytoD treatment reverses the diffusion behaviour of the fast LFA-1 population after SMase treatment closer to untreated cells, strongly suggesting a cross-talk (or interactions) between actin and Cer influencing the mobility of fast LFA-1 nanoclusters.

**Figure 6:**
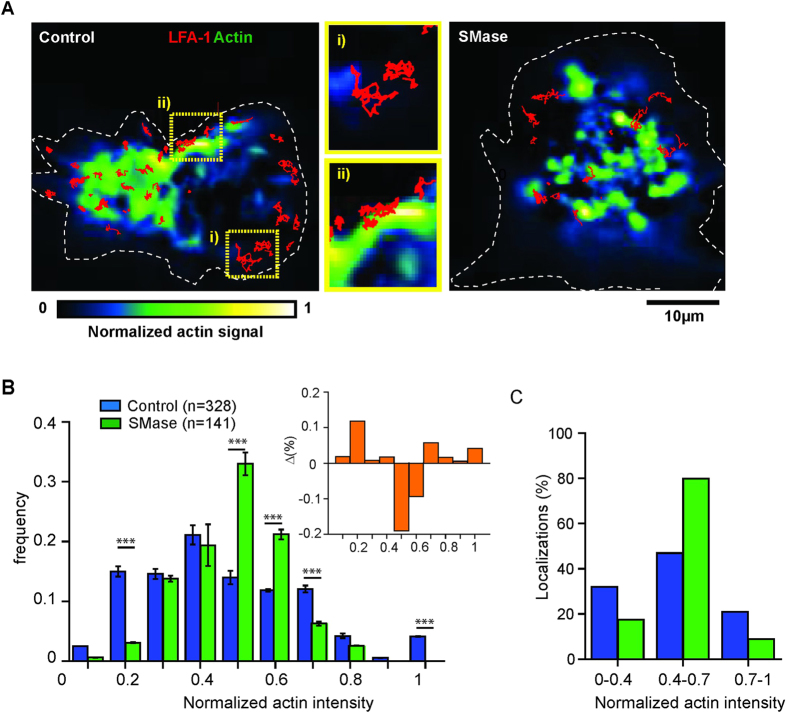
Increased association of LFA-1 with the cytoskeleton upon Cer formation. (**A**) Dual colour images of lifeact-GFP transfected monocytes and SPT of LFA-1 nanoclusters (red trajectories) in control (left) and SMase (right) treated cells. After transfection with lifeact-GFP and, if applicable treatment with SMase, monocytes were labeled with TS2/4-Atto647N and seeded on fibronectin. Movies were recorded at 100ms/frame in TIRF mode. Custom-written software was employed to quantify and normalize the actin signal in each cell by assigning the lowest actin intensity to 0 (blue) and the highest to 1 (yellow), as indicated in the color code (bottom). White dotted lines represent the cell boundaries. Within the control cell, selected area i) represents an example of a low actin signal, while selected area ii) is an example of a high actin signal in control cells, superimposed with the corresponding LFA-1 trajectories (red). Both selected areas are shown as enlarged images next to the control cell. **(B**) Distribution of the normalized actin intensity associated with each LFA-1 trajectory in unperturbed (blue) or SMase treated cells (green). Each histogram contains at least 140 trajectories taken from 15 cells in multiple experiments. The data represents the mean ± SD obtained after bootstrapping of actin intensities measured in all experiments. The inset shows the difference between the normalized frequency of localizations of LFA-1 trajectories in unperturbed cells and upon SMase treatment as function of the normalized actin signal. P-values were compared to unperturbed cells by 2way ANOVA with Bonferroni post-test, ***<0.001. (**C**) Total percentages of LFA-1 trajectory localizations in unperturbed (blue) and SMase treated cells (green) associated with the normalized actin-GFP fluorescent signal for values <0.4, 0.4–0.7 and >0.7 as extracted from B.

To investigate whether formation of Cer induces global changes in actin that could explain the slowing down of LFA-1, we imaged actin after SMase treatment by confocal and TIRF microscopy (Supplementary Fig. S6). While cell stretching was slightly reduced upon SMase treatment with respect to unperturbed cells, confocal and TIRF images did not show global changes in actin structure, labeled with Phalloidin, at the resolution limit of this approach. To determine whether nanoscale changes in the interaction of LFA-1 with cortical actin could occur after SMase treatment, we investigated the association of mobile LFA-1 nanoclusters with actin by simultaneous dual colour imaging of lifeact-GFP in combination with SPT of LFA-1 in TIRF mode. Trajectories of LFA-1 were overlaid on an image of the total actin signal in unperturbed cells as well as cells treated with SMase (Fig. 6A). To account for differences in the actin intensities amongst cells, we normalized the intensities on each image by assigning the highest intensity to 1 and the lowest to 0 (Fig. 6A). To quantify any potential correlation between the spatial location of the LFA-1 trajectories and the actin levels, we used a custom-written software to generate histograms of the normalized actin signal associated with each LFA-1 trajectory (Fig. 6B). Clearly, the distribution of LFA-1 trajectories upon SMase treatment shifted from low towards intermediate actin signal values as compared to untreated cells (Fig. 6B). To substantiate these results we calculated the difference between the normalized frequency of localizations of LFA-1 trajectories from untreated and SMase treated cells at each value of the normalized actin signal (inset Fig. 6B). For actin signals between 0.5–0.6 the residuals are negative so that localizations of LFA-1 trajectories upon SMase treatment are dominant. Below and above 0.5–0.6 the residuals are positive and thus dominated by localisations of untreated LFA-1 trajectories (Fig. 6C). To account for the total percentage of localisations we separated the distributions in three subpopulations according to the actin signal i.e. actin low (signals <0.4), actin intermediate (signals 0.4–0.7) and actin rich (signals ≥0.7) regions (Fig. 6C). In unperturbed cells we found that ~36% of LFA-1 trajectories preferentially localised to actin-low areas (actin index 0.1–0.4) and 46% to intermediate actin-intensities (0.4–0.6), and only a small fraction (~20%) colocalized with actin-rich areas (0.7–1) (Fig. 6C). In strong contrast, on SMase treated cells, a large percentage (80%) of LFA-1 trajectories were found on intermediate actin intensities. These enriched actin regions would then increase the interaction of LFA-1 with the actin cytoskeleton hindering its mobility and explaining therefore the reduction of LFA-1 mobility and its increased anomalous diffusion after SMase treatment.

## Discussion

In this study we investigated the effect of SM reduction on LFA-1 function, cell surface organisation and mobility in monocytes. By combining biochemical assays and extensive SPT and comparing the effect of the metabolic inhibitor myriocin with that of the membrane-associated enzyme SMase, we revealed that conversion of SM into Cer arrests LFA-1 nanocluster mobility and enhances the interactions of LFA-1 with cortical actin prior to ligand binding, thus providing an explanation for the impairment of LFA-1 adhesive function caused by SMase activity.

We have previously shown that on resting monocytes LFA-1 function is associated with the formation of LFA-1 nanoclusters that reside in nanoscale proximity to complexes enriched with raft components such as GM1 and GPI-APs^37^,^38^. LFA-1 function and proximal distribution to raft domains were sensitive to changes in the lipid nano-environment induced by cholesterol depletion via methyl-β-cyclodextrin (MCD)^38^. Since MCD treatment is known to induce global changes in membrane architecture and physiological processes such as signalling^52^, we here used drugs that specifically reduced the SM content, the most common sphingolipid in eukaryotic membranes with an essential role in lipid nanodomain formation^45^. SMase converts SM into Cer by hydrolysis, while myriocin reduces the SM content by metabolic inhibition of the sphingolipid synthesis. In our experiments, SMase or myriocin did not induce significant modifications of PM fluidity or impeded on cell surface availability of integrin receptors. Similar to MCD, SMase and myriocin reduced the proximity of LFA-1 towards GM1-enriched domains and impaired LFA-1 function, while the integrity of LFA-1 nanoclusters and the expression levels of LFA-1 were preserved. As both treatments reduced the SM levels, this suggests that the proximal distribution of LFA-1 to raft domains prior to ligand encounter^3^,^38^ is sensitive to SM levels. While SMase and myriocin both reduced the SM content of the PM, albeit to a different extent, the production of Cer by SMase induces additional molecular changes at the nanoscale, which affected integrin function and mobility differently from myriocin, strongly indicating that these treatments are not interchangeable. In addition, myriocin also affects the levels of GM1, whereas SMase does not. This could actually explain why myriocin inhibits LFA-1 activity although it does not hamper integrin lateral dynamics. Our previous work demonstrated the existence of raft-based interconnectivity at the nanoscale between GPI-AP, LFA-1 and GM1 that forms the basis for large-scale raft coalescence upon cell activation^3^. SM and GM1 are both considered components of lipid rafts and reportedly occupy distinct domains within the PM^5^,^53^. However, GM1 has been shown to interfere with SM phase separation in model membranes^54^, suggesting mutual influence of these glycosphingolipids within the PM. Conversion of SM into Cer will most likely disturb the integrity of GM1 that, through the cholesterol-based connectivity among these domains, might in turn affect LFA-1. Myriocin might affect the nanoscale connectivity between LFA-1 and its PM neighboring partners that we know are important to strengthen nascent adhesion hotspots. Our findings once again strengthen the notion that SMase and myriocin induce different alterations to the PM, and that caution should be used when comparing results of these two conditions.

In contrast to myriocin, SMase lowers SM levels by conversion of SM into Cer, which strongly influences the global organisation and dynamical properties of the PM by the formation of larger Cer-enriched microdomains^21^,^24^,^25^. In addition to the effects on LFA-1 function assigned to a reduction in SM content, the formation of Cer strongly impaired the mobility of LFA-1: SMase activation caused a relative increase of the LFA-1 slow moving sub-population, reduced mobility and increased restrictions on both slow and fast moving sub-populations most likely by increasing the interaction with actin. In addition, a reduction in mobility of LFA-1 by entrapment in Cer domains would decrease the likelihood of LFA-1 to diffuse to the ligand-binding site and to reinforce adhesion, consistent with a recent work showing that integrins cycle through phases of immobilization and diffusion upon ligand binding in focal adhesions^55^. Whether LFA-1 nanoclusters are physically entrapped within Cer platforms needs to be investigated. Although addition of exogenous SM to SMase-treated cells would seem a logical step to rescue PM lipid content and monitor LFA-1 dynamics and function, there is ample evidence in the literature that exogenously added glycosphingolipids are mostly taken up by the cells with only a very minor part being re-inserted into the PM^56^,^57^,^58^. Fluorescently labelled lipids have been exogenously added to the cells to measure their lateral diffusion within the PM^7^,^8^. However, the aim of those experiments however was to obtain as sparse fluorescent lipids as possible within the plasma membrane to be able to perform single molecule measurements by nanoscopy approaches such as STED or NSOM. Under these conditions, the massive internalization of the exogenously added lipids could be neglected as the studies focused on the few fluorescent lipids that managed to stay in the membrane.

Increased receptor clustering induced by Cer has been reported for PM receptors such as the co-stimulatory receptor CD40^59^ and apoptosis initiator CD95^60^, which is a prerequisite for receptor signalling. On the other hand, Cer formation has also been reported to hamper ligand binding capacity of the PM human serotonin1A receptor^61^ while increasing its lateral mobility^62^. Interestingly, in the case of LFA-1, Cer formation does not hamper LFA-1 mediated adhesion by interfering with the integrin nanoscale clustering but rather reduces and restricts LFA-1 diffusion eventually limiting the coalescence of several nanoclusters into stable ligand-binding areas. Future work will determine whether Cer leads to local rearrangements of the cortical actin that actively trap LFA-1 or whether there is a real inter-leaflet connection between Cer platforms and the actin cytoskeleton that overall will influence PM receptor mobility. Clearly, Cer formation affects PM receptors at different levels, and whether the effects are positive or negative for receptor function seems to depend on the type of receptor.

The eukaryotic PM is connected to a dense actin-rich network (cortical actin) which regulates lipid-based PM nanoheterogeneity^49^,^50^ and hinders the diffusion of PM lipids in artificial membranes at physiological temperature^63^. Our simultaneous SPT of LFA-1 and actin revealed increasing association of LFA-1 nanoclusters with cortical actin upon SMase treatment. Potentially, changes in the lipid nanoenvironment upon Cer production alter the interaction of LFA-1 with actin or actin binding proteins, such as Talin and Kindlin-3^64^, which directly connect the cytoplasmic tails of integrins with the cytoskeleton. Interestingly, phospholipids of the inner PM were recently shown to directly influence the affinity of Talin-1 towards integrin β-chain and thereby regulating integrin activation^65^, indicating that integrins are sensitive to changes in their surrounding lipid nanoenvironment. Recent elegant studies by Mayor and colleagues finally demonstrated the existence of PM transbilayer coupling that is important for nanoclustering of GPI-APs, providing the evidence that changes in the lipid content at the PM outer leaflet can be transduced to the inner leaflet^66^.

Several studies have indicated that Cer formation directly influences cortical actin remodeling by altering the cell biomechanical properties^67^, enhancing the assembly of complexes inducing actin polymerization^68^, decreasing cell spreading capacity in measle virus infected T cells^69^ or directly interfering with the interactions between actin and the ERM proteins^70^,^71^. Future studies should address whether Cer formation has direct effect on the cortical actin in the monocytes, thus contributing to the altered interactions between LFA-1 and cortical actin observed here.

Our data are in line with previous findings showing that leukocyte adhesion on neutrophils was impaired upon Cer production, due to a defect of cytoskeletal organization and inside-out signaling^35^. Moreover, we now extend these findings by showing that impaired leukocyte adhesion is likely caused by altered interaction of LFA-1 with the cytoskeleton and a reduction in LFA-1 mobility upon Cer production. Modification of adhesive properties or signaling cascades in immune cells by Cer is highly relevant under physiological conditions. Pathogens, such as measles virus, efficiently induce Cer domains in host T cells, which prevent b1-integrin mediated T cell motility by interference with actin dynamics^51^. Moreover, the sphingolipid and Cer content undergoes changes during cancer, inflammation and auto-immune diseases^19^. Our data indicate that such changes likely affect the adhesive properties of immune cells, which can ultimately impact on the immune response. Sphingolipid play an important role in cell adhesion as they regulate actin-binding proteins such as the ezrin, radixin, and moesin protein families^70^. A study on the effect of the chemotherapeutic cisplatin in cancer cell morphology has given mechanistic insights into the interplay between Cer and the cytoskeleton^71^. Activation of endogenous acid SMase by cisplatin increased the levels of Cer, which in turn activated the serine/threonine phosphatase PP2A that dephosphorylated the actin-binding protein ezrin. As a result, cortical actin dissociated from the PM resulting in the collapse of filopodia and consequently cell motility. LFA-1 adhesion strengthening implies the recruitment of actin-binding proteins to reinforce the ligand-binding site. Although not demonstrated yet, SMase-induced dephosphorylation of actin-binding proteins could contribute to reduced LFA-1 lateral mobility and adhesion by altering the association of the integrin with the cytoskeleton through a yet unknown mechanism.

In summary, this study provides evidence that LFA-1 mobility and function are sensitive to modification of the lipid nanoenvironment within the PM. Our data emphasizes a critical role for Cer in keeping the steady-state diffusion profile of LFA-1 which allows efficient ligand encounter by reinforcement of the binding site by mobile LFA-1 nanoclusters. A dynamically changing lipid content in response to metabolic changes during disease or in response to external stimuli^72^ might represent a so far unexplored mechanism to modulate cell adhesion *in vivo,* potentially exploited by cancer cells with altered adhesive properties, or by pathogens to modulate the adhesive properties of the host cell to escape immune surveillance .

## Materials and Methods

### Antibodies and Reagents

The mouse monoclonal antibodies (mAbs) against the integrin αL chain were NKI-L15^73^, NKI-L16^37^ and TS 2/4 (kindly provided by E. Martz), and the mouse mAb against the β2 chain was L19^73^. For flow cytometry and SPT we included the mAb mouse anti-CD71 (clone B3/25). GM1 was labeled with recombinant Cholera Toxin Subunit B Alexa Fluor® 647-conjugated (Molecular Probes®). To monitor cell viability we used AnnexinV-FITC (BD Pharmingen) and propidium iodide (SIGMA). GPI-APs were detected by fluorescently labeled aerolysin (*FLAER*) (Pinewood Scientific Services Inc.). Secondary stainings were performed with Alexa 488–conjugated goat anti–mouse IgG2a and Alexa 647–conjugated goat anti–mouse IgG1 (both Invitrogen). SM levels were reduced by addition of sphingomyelinase from *Staphylococcus aureus* (Merck, Calbiochem®). For sphingolipid biosynthesis inhibition, myriocin from *Mycelia sterilia* (Sigma-Aldrich) was added during cell culture. Cytochalasin D was purchased from SIGMA. The mAb against LFA-1 (TS2/4) was labelled with Atto647N NHS ester (Fluka) at approximately 1:1 stoichiometry as determined by spectrophotometric measurements (NanoDrop®).

### Cells and drug treatment

THP-1 monocytes were cultured in suspension in RPMI 1640 medium (Gibco) supplemented with 10% fetal calf serum (FCS, from Greiner), antibiotic-antimyotic (AA, from PAA) and ultraglutamine (UG, from Cambrex). One day prior to experiment, cell density was adjusted to a concentration of 0.4 × 10^6^/ml. For SPT studies, cells were cultured in phenol-red free RPMI 1640 medium (Gibco) supplemented with 10% FCS, AA and UG.

For SMase treatment, THP-1 cells were washed in serum-free RPMI 1640 medium and subsequently incubated for 60 min at 37 °C in 0.05U/ml SMase, in the absence of serum. Control cells were treated in the same way except that SMase was not added. For sphingolipid biosynthesis inhibition, cells were cultured in complete medium (containing FCS, AA and UG) for 48 h in the presence of 10 μM myriocin and then serum-starved for 14 to 18 h in the presence of 10 μM myriocin. Cytochalasin D was used at 5 μg/ml and incubated for 5 min at 37 °C prior to measurements.

### Flow Cytometry

For flow cytometry analysis, cells were incubated (30 min, 4 °C) in PBS, 0.5% BSA, and 0.01% sodium azide, with different mAbs (5 μg/ml), followed by incubation with AF647-coupled goat anti-mouse IgG antibody (invitrogen) for 30 min at 4 °C. For GM1 labeling, cells were incubated with CtxAF647 for 20 min at 4 °C. The relative fluorescence intensity was measured on a FACS-Calibur. Isotype-specific controls were included.

### ICAM-1 Fc Fluorescent Bead Adhesion Assay

Carboxylate-modified streptavidin-coated TransFluorSpheres (488/645 nm, 1μmØ; invitrogen) were coated with ICAM-1-Fc, and the bead adhesion assay was performed as described elsewhere 37. Briefly, the fluorescent beads were coated with biotinylated goat anti-human Fc antbodies and subsequently with ICAM-1-Fc chimera. This guarantees the outwards orientation of the ICAM-1 molecules with respect to the bead surface. After each coating step, several thorough washing steps ensure that the excess of unbound molecules is washed away. For the incubation, a ratio of 20 beads per cell was used. The blocking mAbs NKI-L15 was preincubated with the cells before adding the ligand-coated beads. Adhesion was determined as the percentage of cells that bound fluorescent beads by flow cytometry on a FACSCalibur (BD).

### Confocal Microscopy

For co-capping experiments, THP-1 cells were stained at 4 °C with 5 μg/ml anti–LFA-1 mAb (clone NKI-L15 or TS2/4), anti-CD71 and Ctx-AF647. Isotype-specific controls were always included. Following extensive washing steps, patching was induced by incubation with the secondary antibody for 30 min at 4 °C, followed by 1 h incubation at 12 °C. Cells were extensively washed, fixed with 1% PFA and mounted onto poly-l-lysine–coated glass coverslips. Cells were imaged on an Olympus FV1000 Confocal Laser Scanning Microscope and signals were collected sequentially to avoid bleed through. Co-localization of LFA-1 with GM1 was determined by calculating the Manders coefficients M1 (0 = no co-localization and 1 = 100% co-localization) using Image J (http://rsb.info.nih.gov/ij/,) plug-in JACoP^74^.

### Labeling and sample preparation for single particle tracking using fluorescent dyes

Fc-receptors on THP-1 cells were blocked with 1% human serum (HS, from SIGMA) in medium for 5 min at RT, followed by sub-labeling with non-blocking TS2/4-Atto647N in serum free/phenol-red free medium for 5 min at RT. As we were in particular interested to track LFA-1 nanoclusters instead of single LFA-1 heterodimers, we chose whole antibody over Fab fragments for tracking. Cells were extensively washed in serum-free/phenol-red free medium. After washing, cells were seeded for 10 minutes at 37 °C on potassium hydroxide cleaned and sonicated Labtek II chambered cover-glasses (4 well, Nunc), coated with 20ug/ml fibronectin (Roche). Treatment with SMase or myriocin was performed prior to imaging and the drugs remained present for the time of the experiment. CytoD was added to the imaging medium and incubated for 5 min 37 °C prior to imaging.

### Single molecule detection sensitive microscopy set-up

Fluorescence imaging of TS2/4-ATTO647N-labelled LFA-1 was performed on the ventral side of cells using an Olympus IX71 inverted microscope working in total internal reflection fluorescence (TIRF) geometry with a 150×, 1.45NA oil objective. Excitation of ATTO647N was provided by a 640-nm solid-state laser (power density at the focal plane <1 kW/cm^2^). Fluorescence was collected with the same objective and guided into an EM-CCD camera (Hamamatsu ImagEM) after suitable filtering. Movies were recorded at a frame rate of 10 Hz for a total of typically 200 frames. The sample temperature (34–36 °C) was maintained by a stage heater (Pecon) and an objective heater. Under our experimental conditions, the localization precision on the determination of the centroid positions of individual fluorescent spots resulted 25 nm, as assessed from measurements of individual TS2/4-ATTO647N on fixed cells (Fig. S4).

### Fluorescence Trajectory Analysis

Two-dimensional trajectories of individual LFA-1 fluorescent spots in the focal plane were obtained using a custom-made single-particle tracking software (Bakker *et al.*, 2012) Trajectories ≥13 frames were retained for analysis. Individual trajectories were analyzed by calculating the mean-square displacement (MSD) as a function of time lag (t_lag_). Short-range diffusion coefficients were extracted from the linear fit to the 1^st^–4^th^ point of the MSD curve using the following equation MSD = 4D_1–4_t_lag_ + Δ_0_, where *D*_1–4_ is the short range diffusion coefficient and Δ_0_ is the MSD offset at zero time increment. Histograms of all the *D*_1–4_ values per condition were then included in a semilog plot, showing the full distribution of diffusion values. The smallest detectable diffusion coefficient was obtained after imaging TS2/4-Atto647N on fixed cells using TIRFM at the same frame rate as in the corresponding experiments. The 95 percentile of the distribution of short range diffusion constants, which was 0.0046 μm^2^/s for these spots on fixed cells, was considered as the minimum D_1–4_ value for mobile trajectories. In addition, we also generated cumulative MSD plots of all the mobile trajectories per condition and performed linear fitting of the cumulative MSD plot through the first 1–4 points. From the fitting we extracted the average D_1–4_ value of the mobile population. Errors in the D values correspond to the standard deviation to the linear fitting.

### Cumulative Probability Distribution (CPD) Analysis of mobile trajectories

To enquire on the long-term diffusion behavior (>1.5 s) of mobile trajectories, we applied CPD analysis to the data using a similar approach as reported by Bakker *et al.* 2012. In short, mobile trajectories were first selected from the entire dataset as those displaying D_1–4_ > 0.0046 μm^2^∕s. Then the CPD was created for the ensemble of the square displacements (r^2^) of these trajectories at different time lags and fitted with a two-population model describing random diffusion according to Schutz *et al.* 1997:


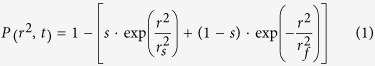


where P (r^2^, t) is the probability that a particle starting at the origin will be found within a circle of radius *r* after time lag t*. s* is the normalized sub-population of slow LFA-1 nanoclusters, (1 − *s*) the normalized sub-population of fast LFA-1 nanoclusters, *r*_*s*_ the square displacement of the slow fraction at timelag t and r_f_ the square displacement of the fast fraction at timelag t. Then, slow and fast sub-population square displacement curves were fitted to derive average short range diffusion coefficient (*D*_1–4_) of each population was determined in a similar manner as described above for individual trajectories. In addition, the square displacement curves corresponding to the slow and fast diffusing populations were also fitted using the anomalous diffusion function (Bakker *et al.*, 2012) to derive the anomalous parameter α for slow and fast LFA-1 populations, where α = 1 corresponds to free, Brownian diffusion and α < 1 corresponds to hindered, anomalous diffusion. Errors in the square displacements were estimated by bootstrapping (resampling residuals approach), the errors bars represent two times the standard deviation originated from fitting procedures.

### Sample preparation for dual colour imaging and SPT experiments

For dual colour single molecule imaging of LFA-1 together with actin, THP-1 cells were transfected with lifeact-GFP by electroporation using the Neon® Transfection device (Invitrogen) and transfection materials (Invitrogen) according to manufacturer´s instructions. Briefly, 2 × 10^6^ cells were transfected with a total of 5ug plasmid DNA. Electroporation was performed at 1300 V, 20 ms and 2 pulses. After electroporation, cells were plated in serum- and antibiotic -free medium for 3 hours at 37 °C. Subsequently, fetal calf serum and antibiotics were added to the medium and cells were cultured for 18 hours. Treatment with SMase was performed as described above.

Dual colour imaging was performed using a custom-built single molecule EPI/TIRF-fluorescence setup. Samples were illuminated in TIRF mode and excitation was provided by a He:Ne laser (633 nm, 4 ms, 1 kW/cm^2^) and an Ar + -Kr + laser (488 nm, 2 ms, ~2 kW/cm^2^). Fluorescence was collected with a 60 × 1.45 NA oil immersion objective (Olympus PLAPO 60 × 0TIRFM NA1.45) and guided into an EM-CCD camera (Hamamatsu). Movies were collected at a frame rate of 10 Hz. Experiments were performed at 37 °C. Simultaneous two-color imaging was achieved by spectrally splitting the Atto647N (red) and lifeact-GFP (green) emissions onto different regions of the EM-CCD camera. Finally, a custom-designed image registration method was used to map the relative positions of the Atto647N and lifeact-GFP over the time course of data acquisition.

### Statistics

The data were compared using 1way ANOVA and Student Neuman–Keuls post-test, or 2way ANOVA with Bonferroni post-test, as indicated. If not highlighted by an asterisk, the results were statistically not significant (ns).

## Additional Information

**How to cite this article**: Eich, C. *et al.* Changes in membrane sphingolipid composition modulate dynamics and adhesion of integrin nanoclusters. *Sci. Rep.* **6**, 20693; doi: 10.1038/srep20693 (2016).

## Supplementary Material

Supplementary Information

## Figures and Tables

**Table 1:** Initial diffusion coefficients, α value and % of slow and fast sub-populations of LFA-1 nanoclusters (imaged by mAb TS2/4 Atto647N) in unperturbed, SMase treated monocytes ± addition of CytoD.

	N	α slow	D_[init]_ slow [μm^2^/s]	α fast	D_[init]_ fast [μm^2^/s]	% immobile	% slow	% fast
Control	658	0.67 ± 0.05	0.0045 ± 4e-05	0.86 ± 0.01	0.053 ± 0.002	21.63 ± 2.37	25.86 ± 1.62	52.51 ± 2.24
SMase	461	0.66 ± 0.04	0.0051 ± 0.003	0.74 ± 0.02	0.035 ± 0.0009	22.06 ± 2.56	31.18 ± 1.83	45.75 ± 1.96
SMase + CytoD	262	1.1 ± 0.06	0.055 ± 7e-05	0.98 ± 0.02	0.049 ± 0.001	21.51 ± 2.04	27.26 ± 2.53	50.66 ± 3.46

**Table 2:** Initial diffusion coefficients, α value, and % of slow and fast sub-populations of LFA-1 nanoclusters (imaged by mAb TS2/4 Atto647N) in unperturbed (cultured in the absence of serum) and myriocin treated monocytes.

	N	α slow	D_[init]_slow [μm^2^/s]	α fast	D_[init]_ fast [μm^2^/s]	% immobile	% slow	% fast
Control (−serum)	467	0.93 ± 0.02	0.0039 ± 0.0002	0.9 ± 0.006	0.042 ± 0.0007	22.32 ± 2.70	20.97 ± 1.79	56.71 ± 2.56
Myriocin	406	0.79 ± 0.06	0.0032 ± 0.0005	0.86 ± 0.01	0.046 ± 0.002	24.58 ± 2.7	23.38 ± 1.98	52.04 ± 2.65

## References

[b1] Cambi A. & Lidke D. S. Nanoscale membrane organization: where biochemistry meets advanced microscopy. ACS Chem Biol 7, 139–149 (2012). 22004174 10.1021/cb200326g PMC3262946

[b2] Lingwood D. & Simons K. Lipid rafts as a membrane-organizing principle. Science 327, 46–50 (2010). 20044567 10.1126/science.1174621

[b3] van Zanten T. S. *et al.* Direct mapping of nanoscale compositional connectivity on intact cell membranes. Proc Natl Acad Sci USA 107, 15437–15442 (2010). 20713733 10.1073/pnas.1003876107 PMC2932581

[b4] Chen Y., Qin J. & Chen Z. W. Fluorescence-topographic NSOM directly visualizes peak-valley polarities of GM1/GM3 rafts in cell membrane fluctuations. J Lipid Res 49, 2268–2275 (2008). 18603643 10.1194/jlr.D800031-JLR200 PMC2533413

[b5] Kiyokawa E. *et al.* Spatial and functional heterogeneity of sphingolipid-rich membrane domains. J Biol Chem 280, 24072–24084 (2005). 15840575 10.1074/jbc.M502244200

[b6] van den Bogaart G. *et al.* Membrane protein sequestering by ionic protein-lipid interactions. Nature 479, 552–555 (2011). 22020284 10.1038/nature10545 PMC3409895

[b7] Eggeling C. *et al.* Direct observation of the nanoscale dynamics of membrane lipids in a living cell. Nature 457, 1159–1162 (2009). 19098897 10.1038/nature07596

[b8] Manzo C., van Zanten T. S. & Garcia-Parajo M. F. Nanoscale fluorescence correlation spectroscopy on intact living cell membranes with NSOM probes. Biophysical journal 100, L8–10 (2011). 21244822 10.1016/j.bpj.2010.12.3690 PMC3021660

[b9] Sahl S. J., Leutenegger M., Hilbert M., Hell S. W. & Eggeling C. Fast molecular tracking maps nanoscale dynamics of plasma membrane lipids. Proc Natl Acad Sci USA 107, 6829–6834 (2010). 20351247 10.1073/pnas.0912894107 PMC2872400

[b10] Zheng H., Liu W., Anderson L. Y. & Jiang Q. X. Lipid-dependent gating of a voltage-gated potassium channel. Nat Commun 2, 250 (2011). 21427721 10.1038/ncomms1254 PMC3072105

[b11] Coskun U., Grzybek M., Drechsel D. & Simons K. Regulation of human EGF receptor by lipids. Proc Natl Acad Sci USA 108, 9044–9048 (2011). 21571640 10.1073/pnas.1105666108 PMC3107302

[b12] Sciacca M. F. *et al.* Two-step mechanism of membrane disruption by Abeta through membrane fragmentation and pore formation. Biophysical journal 103, 702–710 (2012). 22947931 10.1016/j.bpj.2012.06.045 PMC3443794

[b13] Kotler S. A., Walsh P., Brender J. R. & Ramamoorthy A. Differences between amyloid-beta aggregation in solution and on the membrane: insights into elucidation of the mechanistic details of Alzheimer’s disease. Chem Soc Rev 43, 6692–6700 (2014). 24464312 10.1039/c3cs60431d PMC4110197

[b14] Pontier S. M. *et al.* Cholesterol-dependent separation of the beta2-adrenergic receptor from its partners determines signaling efficacy: insight into nanoscale organization of signal transduction. J Biol Chem 283, 24659–24672 (2008). 18566454 10.1074/jbc.M800778200 PMC3259828

[b15] Lingwood D. *et al.* Cholesterol modulates glycolipid conformation and receptor activity. Nat Chem Biol 7, 260–262 (2011). 21460830 10.1038/nchembio.551

[b16] McHenry A. J., Sciacca M. F., Brender J. R. & Ramamoorthy A. Does cholesterol suppress the antimicrobial peptide induced disruption of lipid raft containing membranes? Biochim Biophys Acta 1818, 3019–3024 (2012). 22885355 10.1016/j.bbamem.2012.07.021 PMC3455134

[b17] Tsukamoto M., Kuroda K., Ramamoorthy A. & Yasuhara K. Modulation of raft domains in a lipid bilayer by boundary-active curcumin. Chem Commun (Camb) 50, 3427–3430 (2014). 24396862 10.1039/c3cc47738j PMC3947710

[b18] Brender J. R., McHenry A. J. & Ramamoorthy A. Does cholesterol play a role in the bacterial selectivity of antimicrobial peptides? Front Immunol 3, 195 (2012). 22822405 10.3389/fimmu.2012.00195 PMC3398343

[b19] Maceyka M. & Spiegel S. Sphingolipid metabolites in inflammatory disease. Nature 510, 58–67 (2014). 24899305 10.1038/nature13475 PMC4320971

[b20] Ryland L. K., Fox T. E., Liu X., Loughran T. P. & Kester M. Dysregulation of sphingolipid metabolism in cancer. Cancer Biol Ther 11, 138–149 (2011). 21209555 10.4161/cbt.11.2.14624

[b21] Bollinger C. R., Teichgraber V. & Gulbins E. Ceramide-enriched membrane domains. Biochim Biophys Acta 1746, 284–294 (2005). 16226325 10.1016/j.bbamcr.2005.09.001

[b22] Anderson N. & Borlak J. Drug-induced phospholipidosis. FEBS Lett 580, 5533–5540 (2006). 16979167 10.1016/j.febslet.2006.08.061

[b23] Avota E., Gulbins E. & Schneider-Schaulies S. DC-SIGN mediated sphingomyelinase-activation and ceramide generation is essential for enhancement of viral uptake in dendritic cells. PLoS Pathog 7, e1001290 (2011). 21379338 10.1371/journal.ppat.1001290 PMC3040670

[b24] Chiantia S., Kahya N., Ries J. & Schwille P. Effects of ceramide on liquid-ordered domains investigated by simultaneous AFM and FCS. Biophysical journal 90, 4500–4508 (2006). 16565041 10.1529/biophysj.106.081026 PMC1471841

[b25] Megha & London E. Ceramide selectively displaces cholesterol from ordered lipid domains (rafts): implications for lipid raft structure and function. J Biol Chem 279, 9997–10004 (2004). 14699154 10.1074/jbc.M309992200

[b26] De Tullio L., Maggio B. & Fanani M. L. Sphingomyelinase acts by an area-activated mechanism on the liquid-expanded phase of sphingomyelin monolayers. J Lipid Res 49, 2347–2355 (2008). 18509194 10.1194/jlr.M800127-JLR200

[b27] Hartel S., Fanani M. L. & Maggio B. Shape transitions and lattice structuring of ceramide-enriched domains generated by sphingomyelinase in lipid monolayers. Biophysical journal 88, 287–304 (2005). 15489298 10.1529/biophysj.104.048959 PMC1305007

[b28] Babiychuk E. B., Monastyrskaya K. & Draeger A. Fluorescent annexin A1 reveals dynamics of ceramide platforms in living cells. Traffic 9, 1757–1775 (2008). 18694456 10.1111/j.1600-0854.2008.00800.x

[b29] Grassme H. *et al.* CD95 signaling via ceramide-rich membrane rafts. J Biol Chem 276, 20589–20596 (2001). 11279185 10.1074/jbc.M101207200

[b30] Jia S. J. *et al.* Formation and function of ceramide-enriched membrane platforms with CD38 during M1-receptor stimulation in bovine coronary arterial myocytes. American journal of physiology. Heart and circulatory physiology 295, H1743–1752 (2008). 18723763 10.1152/ajpheart.00617.2008 PMC2593517

[b31] Abdel Shakor A. B., Atia M. M., Kwiatkowska K. & Sobota A. Cell surface ceramide controls translocation of transferrin receptor to clathrin-coated pits. Cellular signalling 24, 677–684 (2012). 22101012 10.1016/j.cellsig.2011.10.016

[b32] Springer T. A. & Dustin M. L. Integrin inside-out signaling and the immunological synapse. Curr Opin Cell Biol 24, 107–115 (2012). 22129583 10.1016/j.ceb.2011.10.004 PMC3294052

[b33] van Kooyk Y. & Figdor C. G. Avidity regulation of integrins: the driving force in leukocyte adhesion. Curr Opin Cell Biol 12, 542–547 (2000). 10978887 10.1016/s0955-0674(00)00129-0

[b34] Kim C., Ye F., Hu X. & Ginsberg M. H. Talin activates integrins by altering the topology of the beta transmembrane domain. The Journal of cell biology 197, 605–611 (2012). 22641344 10.1083/jcb.201112141 PMC3365499

[b35] Feldhaus M. J., Weyrich A. S., Zimmerman G. A. & McIntyre T. M. Ceramide generation *in situ* alters leukocyte cytoskeletal organization and beta 2-integrin function and causes complete degranulation. J Biol Chem 277, 4285–4293 (2002). 11706024 10.1074/jbc.M106653200

[b36] Springer T. A. & Dustin M. L. Integrin inside-out signaling and the immunological synapse. Curr Opin Cell Biol 24, 107–115 (2012). 22129583 10.1016/j.ceb.2011.10.004 PMC3294052

[b37] Cambi A. *et al.* Organization of the integrin LFA-1 in nanoclusters regulates its activity. Molecular biology of the cell 17, 4270–4281 (2006). 16855029 10.1091/mbc.E05-12-1098 PMC1635357

[b38] van Zanten T. S. *et al.* Hotspots of GPI-anchored proteins and integrin nanoclusters function as nucleation sites for cell adhesion. Proc Natl Acad Sci USA 106, 18557–18562 (2009). 19850864 10.1073/pnas.0905217106 PMC2765922

[b39] Bakker G. J. *et al.* Lateral mobility of individual integrin nanoclusters orchestrates the onset for leukocyte adhesion. Proc Natl Acad Sci USA 109, 4869–4874 (2012). 22411821 10.1073/pnas.1116425109 PMC3323969

[b40] Kenworthy A. K. *et al.* Dynamics of putative raft-associated proteins at the cell surface. The Journal of cell biology 165, 735–746 (2004). 15173190 10.1083/jcb.200312170 PMC2172371

[b41] Lenne P. F. *et al.* Dynamic molecular confinement in the plasma membrane by microdomains and the cytoskeleton meshwork. EMBO J 25, 3245–3256 (2006). 16858413 10.1038/sj.emboj.7601214 PMC1523176

[b42] Vrljic M., Nishimura S. Y., Moerner W. E. & McConnell H. M. Cholesterol depletion suppresses the translational diffusion of class II major histocompatibility complex proteins in the plasma membrane. Biophysical journal 88, 334–347 (2005). 15516525 10.1529/biophysj.104.045989 PMC1305010

[b43] Xu Y., Ramu Y. & Lu Z. Removal of phospho-head groups of membrane lipids immobilizes voltage sensors of K+ channels. Nature 451, 826–829 (2008). 18273018 10.1038/nature06618 PMC4026191

[b44] Keizer G. D., Visser W., Vliem M. & Figdor C. G. A monoclonal antibody (NKI-L16) directed against a unique epitope on the alpha-chain of human leukocyte function-associated antigen 1 induces homotypic cell-cell interactions. J Immunol 140, 1393–1400 (1988). 2450126

[b45] Lasserre R. *et al.* Raft nanodomains contribute to Akt/PKB plasma membrane recruitment and activation. Nat Chem Biol 4, 538–547 (2008). 18641634 10.1038/nchembio.103

[b46] Miyake Y., Kozutsumi Y., Nakamura S., Fujita T. & Kawasaki T. Serine palmitoyltransferase is the primary target of a sphingosine-like immunosuppressant, ISP-1/myriocin. Biochem Biophys Res Commun 211, 396–403 (1995). 7794249 10.1006/bbrc.1995.1827

[b47] Horvath A., Sutterlin C., Manning-Krieg U., Movva N. R. & Riezman H. Ceramide synthesis enhances transport of GPI-anchored proteins to the Golgi apparatus in yeast. EMBO J 13, 3687–3695 (1994). 8070398 10.1002/j.1460-2075.1994.tb06678.x PMC395279

[b48] Dustin M. L., Bivona T. G. & Philips M. R. Membranes as messengers in T cell adhesion signaling. Nat Immunol 5, 363–372 (2004). 15052266 10.1038/ni1057

[b49] Chaudhuri A., Bhattacharya B., Gowrishankar K., Mayor S. & Rao M. Spatiotemporal regulation of chemical reactions by active cytoskeletal remodeling. Proc Natl Acad Sci USA 108, 14825–14830 (2011). 21873247 10.1073/pnas.1100007108 PMC3169122

[b50] Goswami D. *et al.* Nanoclusters of GPI-anchored proteins are formed by cortical actin-driven activity. Cell 135, 1085–1097 (2008). 19070578 10.1016/j.cell.2008.11.032 PMC7616455

[b51] Gassert E. *et al.* Induction of membrane ceramides: a novel strategy to interfere with T lymphocyte cytoskeletal reorganisation in viral immunosuppression. PLoS Pathog 5, e1000623 (2009). 19834551 10.1371/journal.ppat.1000623 PMC2757718

[b52] Kabouridis P. S., Janzen J., Magee A. L. & Ley S. C. Cholesterol depletion disrupts lipid rafts and modulates the activity of multiple signaling pathways in T lymphocytes. Eur J Immunol 30, 954–963 (2000). 10741414 10.1002/1521-4141(200003)30:3<954::AID-IMMU954>3.0.CO;2-Y

[b53] Hullin-Matsuda F. & Kobayashi T. Monitoring the distribution and dynamics of signaling microdomains in living cells with lipid-specific probes. Cell Mol Life Sci 64, 2492–2504 (2007). 17876518 10.1007/s00018-007-7281-x PMC11136190

[b54] Bao R., Li L., Qiu F. & Yang Y. Atomic force microscopy study of ganglioside GM1 concentration effect on lateral phase separation of sphingomyelin/dioleoylphosphatidylcholine/cholesterol bilayers. J Phys Chem B 115, 5923–5929 (2011). 21526782 10.1021/jp2008122

[b55] Rossier O. *et al.* Integrins beta1 and beta3 exhibit distinct dynamic nanoscale organizations inside focal adhesions. Nat Cell Biol 14, 1057–1067 (2012). 23023225 10.1038/ncb2588

[b56] Chigorno V. *et al.* Sphingolipid uptake by cultured cells: complex aggregates of cell sphingolipids with serum proteins and lipoproteins are rapidly catabolized. J Biol Chem 280, 2668–2675 (2005). 15548542 10.1074/jbc.M407749200

[b57] Tomas M. *et al.* Fluorescent analogues of plasma membrane sphingolipids are sorted to different intracellular compartments in astrocytes; Harmful effects of chronic ethanol exposure on sphingolipid trafficking and metabolism. FEBS Lett 563, 59–65 (2004). 15063723 10.1016/S0014-5793(04)00245-5

[b58] Watanabe R., Asakura K., Rodriguez M. & Pagano R. E. Internalization and sorting of plasma membrane sphingolipid analogues in differentiating oligodendrocytes. J Neurochem 73, 1375–1383 (1999). 10501180 10.1046/j.1471-4159.1999.0731375.x

[b59] Grassme H., Jendrossek V., Bock J., Riehle A. & Gulbins E. Ceramide-rich membrane rafts mediate CD40 clustering. J Immunol 168, 298–307 (2002). 11751974 10.4049/jimmunol.168.1.298

[b60] Grassme H., Schwarz H. & Gulbins E. Molecular mechanisms of ceramide-mediated CD95 clustering. Biochem Biophys Res Commun 284, 1016–1030 (2001). 11409897 10.1006/bbrc.2001.5045

[b61] Paila Y. D., Ganguly S. & Chattopadhyay A. Metabolic depletion of sphingolipids impairs ligand binding and signaling of human serotonin1A receptors. Biochemistry 49, 2389–2397 (2010). 20170167 10.1021/bi1001536

[b62] Ganguly S., Paila Y. D. & Chattopadhyay A. Metabolic depletion of sphingolipids enhances the mobility of the human serotonin1A receptor. Biochem Biophys Res Commun 411, 180–184 (2011). 21726540 10.1016/j.bbrc.2011.06.127

[b63] Honigmann A. *et al.* A lipid bound actin meshwork organizes liquid phase separation in model membranes. Elife 3, e01671 (2014). 24642407 10.7554/eLife.01671 PMC3957580

[b64] Hogg N., Patzak I. & Willenbrock F. The insider’s guide to leukocyte integrin signalling and function. Nat Rev Immunol 11, 416–426 (2011). 21597477 10.1038/nri2986

[b65] Moore D. T. *et al.* Affinity of talin-1 for the beta3-integrin cytosolic domain is modulated by its phospholipid bilayer environment. Proc Natl Acad Sci USA 109, 793–798 (2011). 22210111 10.1073/pnas.1117220108 PMC3271903

[b66] Raghupathy R. *et al.* Transbilayer lipid interactions mediate nanoclustering of lipid-anchored proteins. Cell 161, 581–594 (2015). 25910209 10.1016/j.cell.2015.03.048 PMC4651428

[b67] Babahosseini H., Roberts P. C., Schmelz E. M. & Agah M. Bioactive sphingolipid metabolites modulate ovarian cancer cell structural mechanics. Integr Biol (Camb) 5, 1385–1392 (2013). 24056950 10.1039/c3ib40121a PMC3908782

[b68] Park S. S. *et al.* C(16)-Ceramide-induced F-actin regulation stimulates mouse embryonic stem cell migration: involvement of N-WASP/Cdc42/Arp2/3 complex and cofilin-1/alpha-actinin. Biochim Biophys Acta 1831, 350–360 (2013). 22989773 10.1016/j.bbalip.2012.09.005

[b69] Mueller N., Avota E., Collenburg L., Grassme H. & Schneider-Schaulies S. Neutral sphingomyelinase in physiological and measles virus induced T cell suppression. PLoS Pathog 10, e1004574 (2014). 25521388 10.1371/journal.ppat.1004574 PMC4270778

[b70] Adada M., Canals D., Hannun Y. A. & Obeid L. M. Sphingolipid regulation of ezrin, radixin, and moesin proteins family: implications for cell dynamics. Biochim Biophys Acta 1841, 727–737 (2014). 23850862 10.1016/j.bbalip.2013.07.002 PMC3888837

[b71] Zeidan Y. H., Jenkins R. W. & Hannun Y. A. Remodeling of cellular cytoskeleton by the acid sphingomyelinase/ceramide pathway. The Journal of cell biology 181, 335–350 (2008). 18426979 10.1083/jcb.200705060 PMC2315679

[b72] Ma D. W. Lipid mediators in membrane rafts are important determinants of human health and disease. Appl Physiol Nutr Metab 32, 341–350 (2007). 17510668 10.1139/H07-036

[b73] Keizer G. D. *et al.* Biochemical and functional characteristics of the human leukocyte membrane antigen family LFA-1, Mo-1 and p150, 95. Eur J Immunol 15, 1142–1148 (1985). 2933266 10.1002/eji.1830151114

[b74] Bolte S. & Cordelieres F. P. A guided tour into subcellular colocalization analysis in light microscopy. Journal of microscopy 224, 213–232 (2006). 17210054 10.1111/j.1365-2818.2006.01706.x

