# Supplementary material for: Changes in membrane sphingolipid composition modulate dynamics and adhesion of integrin nanoclusters
Source: Sci Rep. 2016 Feb 12;6:20693. doi: 10.1038/srep20693 (PMC4751618; doi:10.1038/srep20693)
Supplement: Supplementary Information [file srep20693-s1.pdf]

Changes in membrane sphingolipid composition modulate dynamics and adhesion of integrin nanoclusters

Running title:

Membrane lipids modulate integrins

Christina Eich[1,2], Carlo Manzo[3,] Sandra de Keijzer[1], Gert-Jan Bakker[4], Inge Reinieren-Beeren[1], Maria F. García-Parajo[3,5], Alessandra Cambi[1]*

[1]Department of Tumor Immunology, Radboud Institute for Molecular Life Sciences, Radboud University Medical Centre, Postbox 9101, 6500 HB Nijmegen, The Netherlands

[2]current address: Erasmus MC Stem Cell Institute, Erasmus MC, Wytemaweg 80, 3015 CN Rotterdam, the Netherlands

[3]ICFO-Institut de Ciencies Fotoniques, Mediterranean Technology Park, 08860 Castelldefels (Barcelona), Spain.

[4] Department of Cell Biology, Radboud Institute for Molecular Life Sciences, Radboud University Medical Centre, Postbox 9101, 6500 HB Nijmegen, The Netherlands

[5]ICREA-Institució Catalana de Recerca i Estudis Avançats, 08010 Barcelona, Spain.

*Corresponding author:

Alessandra Cambi, Dr.

PO Box 9101

6500 HB Nijmegen, the Netherlands

Tel: +31 (0)24 3617600

Fax: +31 (0)24 3540339

E-mail: alessandra.cambi@radboudumc.nl

Supplementary Figures

***Eich et al* Supplementary Fig S1.**

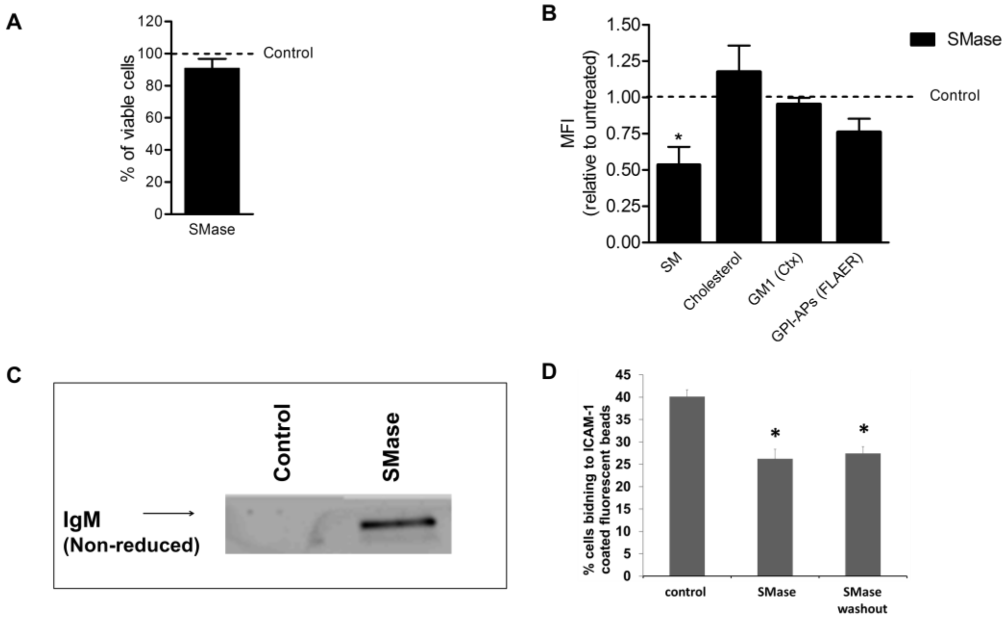

***Eich et al.* Supplementary Fig. S2.**

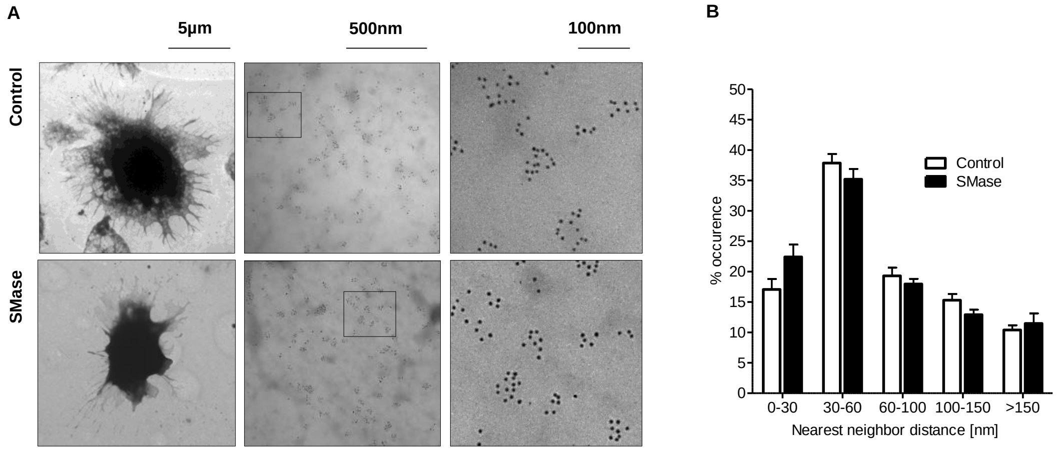

Eich et al **Supplementary Fig. S3.**

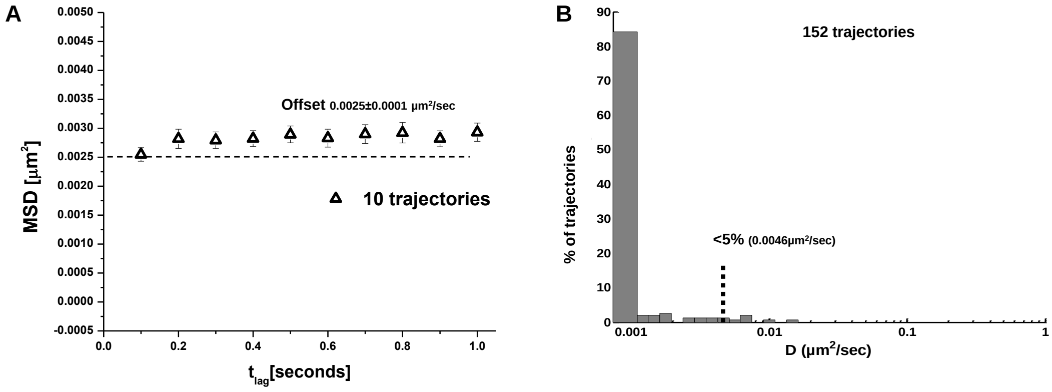

Eich et al. **Supplementary Fig. S4.**

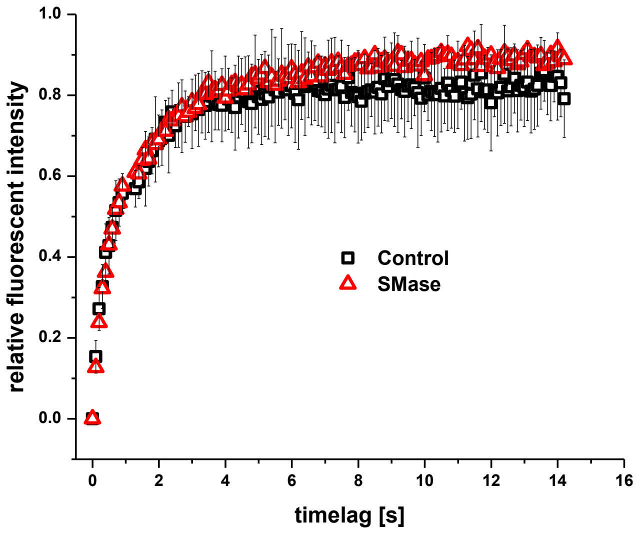

Eich et al. **Supplementary Fig. S5.**

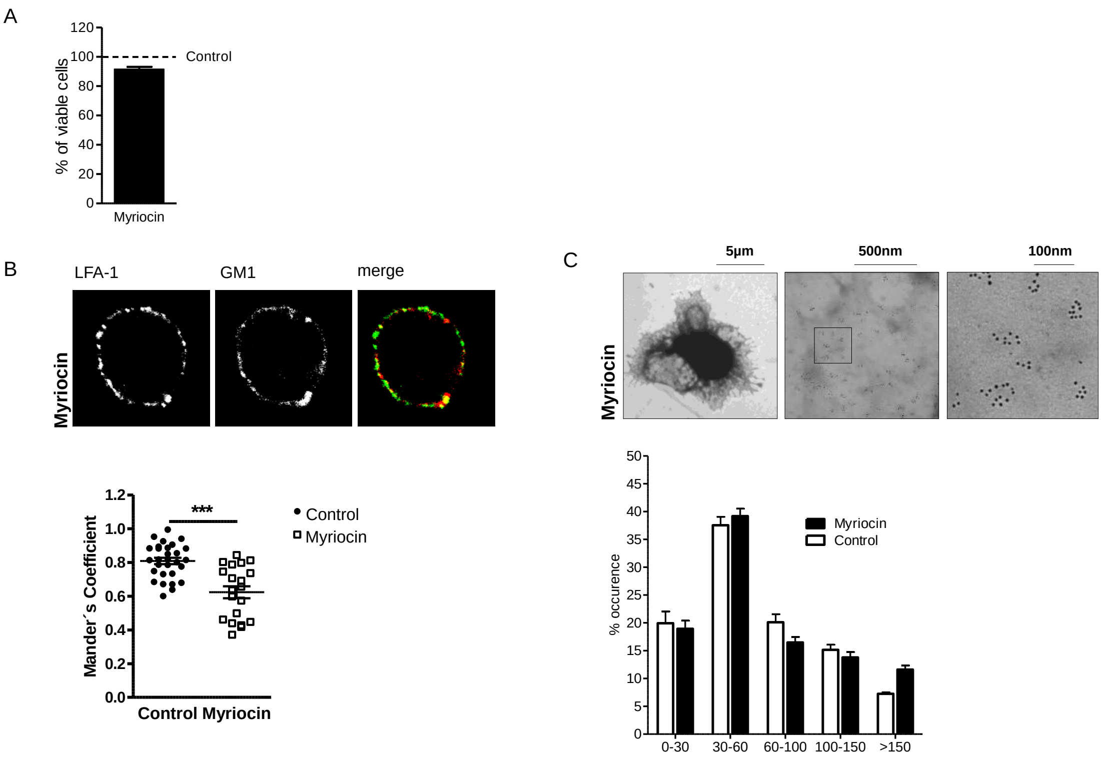

Eich et al. **Supplementary Fig. S6.**

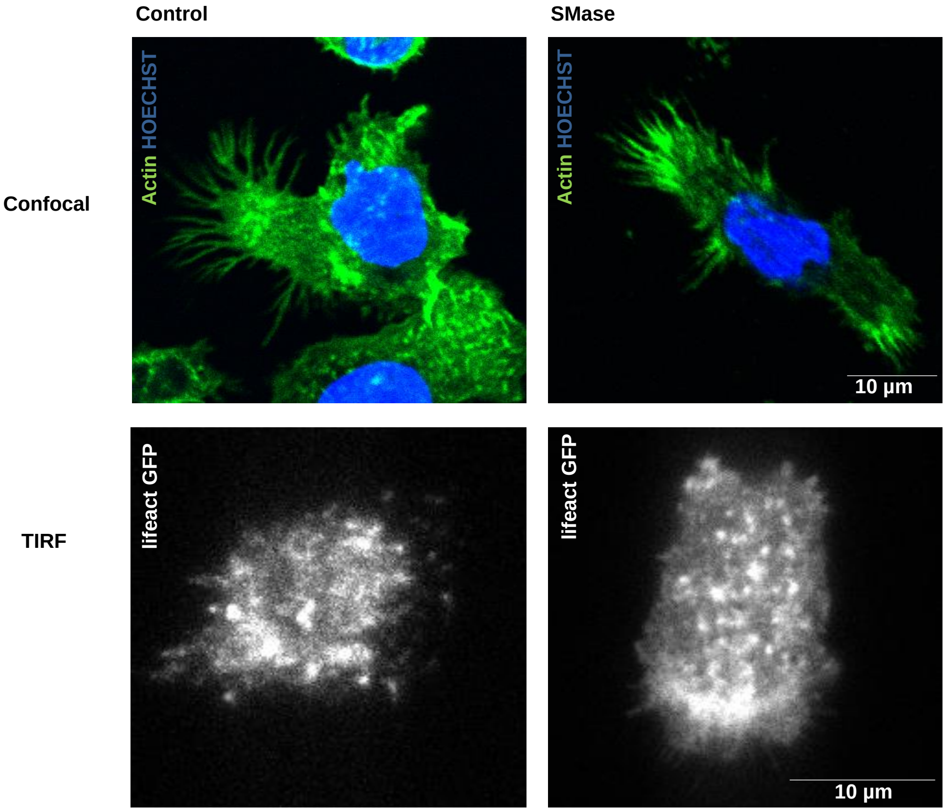

Supplementary Figure Legends

Supplementary Fig. S1. SMase treatment did not affect cell viability, but reduced the PM SM content. (A) Viability of monocytes was not affected by SMase treatment. After 1 hour treatment of monocytes (THP-1 cells) with SMase (0.05U/ml), see *Materials and Methods*, AnnexinV-FITC and propidium iodide (PI) were added to determine the percentage of apoptosis and necrosis, respectively. PI/AnnV negative cells represent the live cell population. Changes in cell viability are displayed as relative to the respective unperturbed samples (expression levels in control cells were set as = 100%). **(**B) PM SM content was determined by labeling of intact cells with lysenin at 4ºC. Extrafacial cellular cholesterol levels were determined on intact cells after SMase treatment by filipin III labeling, PM GM1 levels were detected by Ctx-AF647 and PM GPI-APs were detected by fluorescently labeled aerolysin (*FLAER*). The relative expression was assessed by flow cytometry and changes in the mean fluorescent intensity are displayed as relative to the respective unperturbed sample (expression levels in untreated cells were set as = 1, indicated by the dotted line). The data represent the mean +/- SEM of 3-5 independent experiments. **(**C) Induction of Cer by SMase was determined in unperturbed and SMase treated cells by dissociation of cell surface bound anti-Ceramide antibody, followed by SDS-PAGE and detection of dissociated antibody by WB labeling. While in unperturbed cells extrafacial ceramide was not detectable, conversion of SM into Cer by SMase led to a clear increase in anti-Cer bound antibody. (D) Adhesion of human monocytes (THP-1) was determined using ICAM-1-Fc coated fluorescent beads at 37°C in unperturbed cells, upon treatment with SMase (0.05U/ml) followed or not by washout and 30min recover in warm medium. The % of adhesion represents the amount of cells that have bound beads as determined by flow cytometry. The data

shows one representative experiment performed in duplicate out of 2 ± SD. Differences were assessed by 1way ANOVA test, * <0.05.

Supplementary Fig. S2. LFA-1 nanoclusters were not affected by SMase treatment. Monocytes were specifically labeled for LFA-1 with 10-nm gold for TEM (see Materials and Methods), in unperturbed cells and after treatment with SMase. (A) (left) Representative TEM image of monocytes left unperturbed or treated with SMase. (middle) Overview of the PM of unperturbed or SMase-treated monocytes. 10-nm gold particles are visible. (right) Magnification of an area in the PM highlighted in the middle panel. Nanoclusters of LFA-1 are visible in both conditions. (B) Gold labels were counted and coordinates were assigned to each feature. The interparticle distances were calculated using a nearest neighbor (nn) distance algorithm by custom-written software. nn distance values were calculated for each image. Subsequently, the nn distances were divided into three classes: 0–50, 50–100, and 100 nm, and the percentage of nn distance values falling into each class were plotted in unperturbed cells and upon SMase treatment.

Supplementary Fig. S3. Positional accuracy and determination of the minimal diffusion coefficient. (A) Positional accuracy. Averaged MSD curves of TS2/4-AF647 fluorescent spots on fixed monocytes. 10 immobile trajectories. **(**B) Determination of the minimal diffusion coefficient. Normalized diffusion histograms of TS2/4- Atto647 sub-labeled LFA-1 nanoclusters on fixed cells for a trajectory length of 13 frames. After appropriate labeling, cells were fixed with 3% PFA in PBS. The minimum diffusion coefficient was retrieved by a linear fit through the 4 first

points of the MSD plots of 152 immobile trajectories with a length ≥ 13 frames. The minimum detectable diffusion coefficient of 0.0046µm^2/s was determined with 95% certainty.

Supplementary Fig. S4. SMase treatment did not induce global changes in the PM. THP-1 cells were treated with SMase (0.05U/ml) prior and during the experiment. The membrane was labeled with green fluorescent PKH dye to determine the mobility of membrane lipids after SMase treatment by FRAP. FRAP curves were averaged from 12-15 curves of 2-3 independent experiments and were acquired at 37°C. The error bars represent the SD.

Supplementary Fig. S5. Myriocin did not affect cell viability and LFA-1 clustering, but reduced GM1-LFA-1 co-distribution. (A) Viability of monocytes was not affected by Myriocin treatment. After 3 days of incubation with myriocin (10µM), see *Materials and Methods*, AnnexinV-FITC and propidium iodide (PI) were added to determine the percentage of apoptosis and necrosis, respectively. PI/AnnV negative cells represent the live cell population. Changes in cell viability are displayed as relative to the respective unperturbed samples (expression levels in untreated cells were set as = 100%). **(**B) (top) Confocal microscopy analysis of co-capping of LFA-1 (L15 labeled) and GM1 (CTx-AF647). Receptor co-capping and staining were performed as described in *Material and Methods* in unperturbed cells, or after treatment with myriocin. (bottom) Manders coefficient quantifying the degree of co-localization between LFA-1 and GM1. M1 can vary between 0 and 1 (1 = 100% co-localization). Results are representative of multiple cells in three independent experiments). All P-values were compared to the respective unperturbed cells by

1way ANOVA with Bonferroni post-test, *** <0.001. (C) Monocytes were specifically labeled for LFA-1 with 10-nm gold for TEM (see Materials and Methods), in unperturbed cells and after treatment with myriocin. (top) Representative TEM images of monocytes treated with myriocin. (bottom) Gold labels were counted and coordinates were assigned to each feature. The interparticle distances were calculated using a nearest neighbor (nn) distance algorithm. The nn distances were divided into three classes: 0–50, 50–100, and 100 nm, and the percentage of nn distance values falling into each class were plotted in unperturbed cells and upon SMase treatment.

Supplementary Fig. S6. SMase treatment did not induce dramatic changes in the actin cytoskeleton. (top) Confocal images of unperturbed (left) or SMase treated (right) monocytes seeded on fibronectin, followed by fixation and labeling with Phalloidin-Texas Red and Hoechst. (bottom) TIRF images of lifeact-GFP transfected monocytes, unperturbed (left) or treated with SMase (right), seeded on Fibronectin.

Supplementary Materials and Methods

Detection of ceramide, sphingomyelin and cholesterol

For detection of cell surface ceramides, we adopted an assay as previously described [1]. Briefly, $3x10^6$ THP-1 cells (unperturbed or SMase treated) were fixed with 1% formaldehyde and incubated for 2 hours at RT with α-ceramide IgM (clone MID 15B4, Alexis). Cell bound antibody was desorbed for 30 seconds with ice cold 100mM glycine-HCl, pH 2,5 and subsequently neutralized with 100 mM Tris-HCl pH 8.0. Desorbed antibody was separated by SDS-PAGE and transferred to a PVDF membrane (Millipore, Bedford, MA). Anti-ceramide antibody was detected by goat-anti-mouse IgM coupled to AF647 (Invitrogen). Western blots were scanned by the Odyssey imager (LI-COR Biosciences).

The SM content was detected indirectly by lysenin protein isolated from the earthworm *Eisenia foetida* (Peptanova). Briefly, unperturbed, SMase or myriocin treated THP-1 cells were fixed with 3% formaldehyde, washed with PBS and subsequently quenched with 50mM NH4Cl. Cells were permeabilized with 50ug/ml digitonin, followed by blocking with 0.2% gelatine. 2x10e5 THP-1 cells were incubated with 1ug/ml lysenin in PBA and incubated for 1 hour on ice. Cell- absorbed lysenin was detected by rabbit anti-lysenin antiserum (Peptanova), followed by secondary antibody labelling with donkey anti-rabbit (heavy + light chain) conjugated to AF647 (Invitrogen). The relative fluorescence intensity was measured on a FACS-Calibur.

Extra facial cholesterol was detected by Filipin III from *Streptomyces filipinensis* (Sigma-Aldrich). Briefly, filipin III was reconstituted with DMSO prior to labelling. Unperturbed, SMase or myriocin treated THP-1 cells were fixed with 3% formaldehyde, washed with PBS and subsequently quenched with 1.5mg/ml glycine

in PBS. THP-1 cells were labelled for 30 min at RT with a working solution of 0.05mg/ml filipin III in PBS containing 10% FCS. Subsequently, cells were washed in PBS and fixed in 1% PFA. Cell bound Filipin-III was excited with a UV laser and detected with a 450/450 filter set, measured on a Beckton Coulter EPICS©Altra™ FACS sorter.

Fluorescence Recovery after Photobleaching (FRAP)

FRAP of green fluorescent PKH dye was performed on a LSM510 meta confocal laser scanning microscope (Zeiss, Germany) with a 63x, 1.45NA oil objective. AF488 fluorescence was excited at 488 nm (argon laser), while the emission was collected with 500-550nm bandpass filter adjusted through mirrors. FRAP experiments were performed using a 2 μm diameter circular region of interest in the basal plane of the plasma membrane. Photobleaching was performed operating at 100% of laser power by scanning the bleached ROI for 2 iterations, yielding in a total bleach time of 0.10 s and an average fluorescence loss of ~50%. Recoveries were collected at time intervals of 100 ms. Fluorescence intensity values of the bleached area, as well of an unbleached area of similar size within the same cell, were exported from the Zeiss software and analyzed in Origin. After background correction and normalization to t0 using a method that is known as double normalization [2], the data file (approximately 10-15 curves for each condition) was averaged to create a single curve. The single postbleached curves were fitted with the following model:

$I(t) = y_0 + A_1 e^{(-t/\tau_1)} + A_2 e^{(-t/\tau_2)}$ were y_0 is the mobile fraction and A_1 and A_2 are the fraction sizes of the two subpopulations. The halftime recovery values were calculated with $t_{1/2} = \ln 2 * \tau$.

Electron Microscopy Labeling Procedure

For transmission electron microscope analysis of whole-mount samples, THP-1 cells were allowed to spread on glass coverslips covered by a thin layer of poly-l-lysine (PLL)-coated Formvar for 15 min at 37°C, washed to remove unbound cells, and immediately fixed with 1% PFA for 15 min at RT. When SMase or myriocin were applied, drug treatment was performed prior to seeding cells on PLL-coated coverslips. Despite SMase or myriocin treatment, monocytes were able to stretch on PLL . Subsequent Ab and gold labeling was performed as reported elsewhere [3]. Briefly, After two washing steps with PBS and a subsequent incubation (60 min at room temperature) with blocking buffer (PBS, 0.1% glycine, 1% BSA, and 0.25% gelatin) to reduce specific background, the specimens were incubated for 30 min with primary antibodies against αL (TS2/4) in blocking buffer on ice, rinsed in PBS, and fixed in 1% paraformaldehyde and 0.1% glutaraldehyde for 15 min. After two washing steps with PBS and blocking buffer, the samples were incubated with rabbit anti-mouse IgG (to detect mAb) for 30 min on ice. A final incubation with 10-nm diameter gold-labeled Protein A (to detect polyclonal antibodies) was performed, followed by final fixation in 1% glutaraldehyde in phosphate buffer for 20 min at room temperature. After gold labeling and fixation, the specimens were dehydrated and transferred from the glass onto copper grids as already published [3].

Analysis of Gold Particle Distribution Pattern

The specimens were observed in a JEOL 1010 transmission electron microscope (Welwyn Garden City, United Kingdom), operating at 60–80 kV. Because THP-1 widely spread, the membrane available for gold particle analysis represented up to

60–70% of the whole labeled plasma membrane. For each cell several areas were analyzed at random. The digital images of electron micrographs were processed by custom-written software based on Labview (National Instruments, Austin, TX) as already described [3].

Actin staining of monocytes

Texas Red-conjugated phalloidin (Invitrogen) was used to stain F-actin.

References

1 Korzeniowski, M. *et al.* Fc gamma RII activation induces cell surface ceramide production which participates in the assembly of the receptor signaling complex. *Cell Physiol Biochem* **20**, 347-356.

2 Phair, R. D., Gorski, S. A. & Misteli, T. Measurement of dynamic protein binding to chromatin in vivo, using photobleaching microscopy. *Methods Enzymol* **375**, 393-414.

3 Cambi, A. *et al.* Microdomains of the C-type lectin DC-SIGN are portals for virus entry into dendritic cells. *J Cell Biol* **164**, 145-155.
